# Probing Temperature Responsivity of Microgels and Its Interplay with a Solid Surface by Super-Resolution Microscopy and Numerical Simulations

**DOI:** 10.1021/acsnano.2c07569

**Published:** 2023-01-19

**Authors:** Xhorxhina Shaulli, Rodrigo Rivas-Barbosa, Maxime J. Bergman, Chi Zhang, Nicoletta Gnan, Frank Scheffold, Emanuela Zaccarelli

**Affiliations:** † Department of Physics, University of Fribourg, Chemin du Musée 3, 1700Fribourg, Switzerland; ‡ Department of Physics, Sapienza University of Rome, Piazzale Aldo Moro 2, 00185Roma, Italy; § CNR Institute of Complex Systems, Uos Sapienza, Piazzale Aldo Moro 2, 00185Roma, Italy

**Keywords:** microgels, super-resolution fluorescence microscopy, modeling, solid−liquid interface, volume phase transition

## Abstract

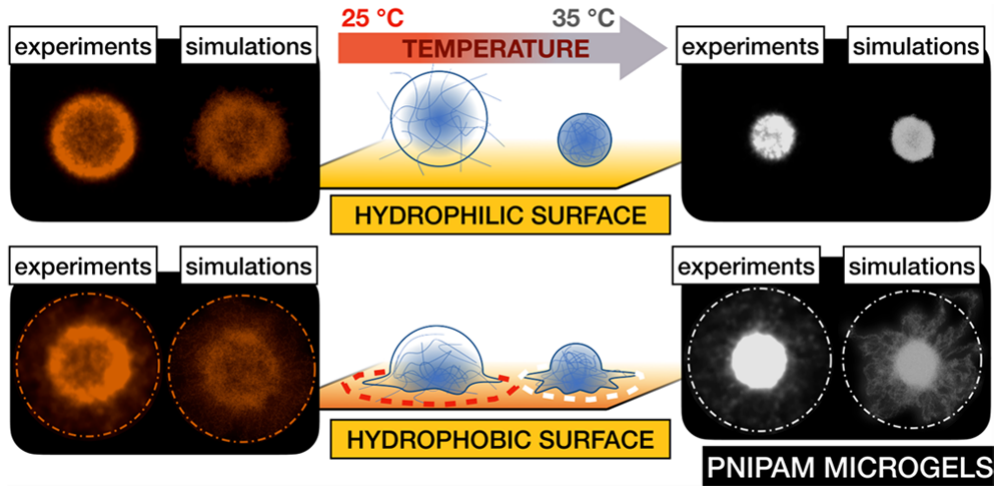

Super-resolution microscopy has become a powerful tool to investigate the internal structure of complex colloidal and polymeric systems, such as microgels, at the nanometer scale. An interesting feature of this method is the possibility of monitoring microgel response to temperature changes *in situ*. However, when performing advanced microscopy experiments, interactions between the particle and the environment can be important. Often microgels are deposited on a substrate, since they have to remain still for several minutes during the experiment. This study uses direct stochastic optical reconstruction microscopy (dSTORM) and advanced coarse-grained molecular dynamics simulations to investigate how individual microgels anchored on hydrophilic and hydrophobic surfaces undergo their volume phase transition with temperature. We find that, in the presence of a hydrophilic substrate, the structure of the microgel is unperturbed and the resulting density profiles quantitatively agree with simulations performed under bulk conditions. Instead, when a hydrophobic surface is used, the microgel spreads at the interface and an interesting competition between the two hydrophobic strengths,monomer–monomer vs monomer–surface,comes into play at high temperatures. The robust agreement between experiments and simulations makes the present study a fundamental step to establish this high-resolution monitoring technique as a platform for investigating more complex systems, these being either macromolecules with peculiar internal structure or nanocomplexes where molecules of interest can be encapsulated in the microgel network and controllably released with temperature.

## Introduction

One of the fascinating aspects of colloidal science is the possibility of investigating mesoscopic particles with experimental techniques that are able to resolve their structure and dynamics at the single-particle level. Thanks to real-space particle tracking, colloidal particles with sizes ranging from hundreds of nanometers up to a few micrometers have long been used as suitable benchmarks to test theories and numerical models. This approach has been successful for a long time, bringing important experimental contributions to fundamental problems such as, for example, the nucleation and growth of colloidal crystals,^1^ the structural relaxation dynamics close to the glass transition,^2,3^ or the 2D melting scenario of hard colloids.^4^ Recently, however, the rapidly growing field of “smart” materials, i.e. materials designed to respond to external stimuli, has moved the community’s attention from standard “hard-sphere” colloids to softer particles, mainly of polymeric nature and with a complex internal architecture. Within this category microgels are among the most studied colloidal systems. Microgels are cross-linked polymer networks whose properties are intimately related to the type of polymer they are made of and to the topology of the network. Their complex architecture provides particles with an internal elasticity that allows them to shrink, deform, and interpenetrate to some extent. Even more intriguing is their ability to respond to external stimuli: depending on the type of polymer employed in the synthesis, microgels can adjust their size in response to temperature, light, or pH changes, to name a few.^5−7^ A well-known example is that of poly(*N*-isopropylacrylamide) (pNIPAM)-based microgels that exhibit conformational changes in response to solvent composition or temperature. Specifically, they undergo a reversible volume phase transition (VPT) at a temperature of about 32 °C that leads to network shrinking and, consequently, to a change in the size of the particle.^8−11^ Additionally, it is worth stressing that synthesis advances allow tuning of the microgel size and architecture, as well as decoration and functionalization, all of which in the end influence the responsiveness to external cues^9,12^ and the mutual effective interactions between the particles.^13^

All these features provide microgels with a richer behavior compared to standard “hard-sphere” colloids: in particular, the possibility to tune their properties in a controllable way makes them suitable as building blocks for designing materials that can be used for several purposes, from understanding fundamental problems in physics^9,14−17^ to industrial applications such as, for example, viscosity modifiers, tunable optical scattering components, or carrier agents.^18−20^ In this context, understanding how the internal complexity of microgels at the nanoscale translates into specific macroscopic material properties is one of the main goals of soft-matter physics. This requires the use of experimental techniques that go beyond standard optical microscopy and push the resolution below the microscale.

To this aim, the recent scientific and technological breakthrough in super-resolved fluorescence microscopy (SRFM)^21−23^ has had a direct impact on many research areas in biology and materials science. These advanced techniques were soon embraced as valuable characterization tools with high accuracy and specificity using multicolor fluorescent labeling protocols.^24−26^ Compared to biological studies, applications of SRFM progressed more slowly in investigating colloidal and polymer systems. More recently, however, it has been shown to be a valuable imaging and characterization method.^27−32^ In contrast to other characterization methods, such as X-ray scattering or atomic force microscopy (AFM), SRFM provides single-particle information on the nanoscale and chemical specificity at the same time.

In the past years, SRFM combined with other methods, like computer simulations and light scattering techniques, have been used to tackle many unanswered questions regarding the microgel network and its properties. In particular, Conley et al. demonstrated the successful application of direct stochastic optical reconstruction microscopy (dSTORM) to investigate stimuli-responsive pNIPAM microgels^28^ at different solvent compositions. The same technique was then used in pure water by the same authors to probe the behavior of concentrated microgels suspensions under swollen conditions.^26^ In addition, Bergmann et al. studied microgels with different cross-linking densities using dSTORM via a nonspecific labeling approach.^33,34^ Different kinds of microgels were studied: first, Wöll and co-workers investigated core–shell microgel particles combining *in situ* electron and super-resolution microscopy to unravel structural details of their particles,^30^ then they studied poly (N-isopropylmethacrylamide) (pNIPMAM) microgels on solid–liquid interfaces below the VPT, showing the dependence of the microgel spread with the surface degree of hydrophobicity^35^ and with the procedure in which the microgel is placed on it.^36^ None of these previous super-resolution studies have tackled the problem of probing the thermoresponsivity of the microgels directly by changing temperature, in order to directly visualize the occurrence of the VPT in the internal structure of the microgels. The only notable exception is the application of the PAINT (Points Accumulation for Imaging in Nanoscale Topography) technique to core–shell pNIPAM-pNIPMAM microgels,^37^ which however only gained information on the radial distribution of the polarity within the particles, rather than the true polymer density profile.

Here we fill this gap by providing a dSTORM investigation of the volume phase transition of pNIPAM microgels, validating the experimental approach through a comparison with state-of-the-art computer simulations. Indeed, the application of the dSTORM technique currently requires the anchoring of the microgels at a nearby surface that, in principle, could affect the results. The present work shows that the use of a hydrophilic surface allows us to probe the VPT without detectable external perturbation of the microgel structure, both in the swollen and in the collapsed state. This is achieved by a careful comparison with numerical simulations both in the presence and in the absence of the nearby surface. Next, we actually exploit the presence of the surface to also characterize the VPT of a microgel close to a hydrophobic wall, thus revealing a subtle interplay between surface adhesion energy and hydrophobic interactions within the microgel, whose microscopic mechanism is unveiled by the numerical simulations. Our work thus represents a crucial step in the nanoscopic characterization of thermoresponsive soft particles.

## Results

### dSTORM Imaging of the Volume Phase Transition of Microgels

We investigate pNIPAM microgels with ∼1.5 mol % BIS using dSTORM across the volume phase transition. Particles are fluorescently labeled in the outer region to facilitate discrimination of different parts of the microgel, namely the core region and the shell including the dangling ends: i.e., the microgel corona. Details on the labeling protocol can be found in Materials and Methodology. In the following, we denote as a *shell* the part of the microgel network that is dye-labeled, even if the transition from the dense core to the loosely cross-linked corona is rather gradual and there is no sharp border.^10,32^

**Figure 1:**
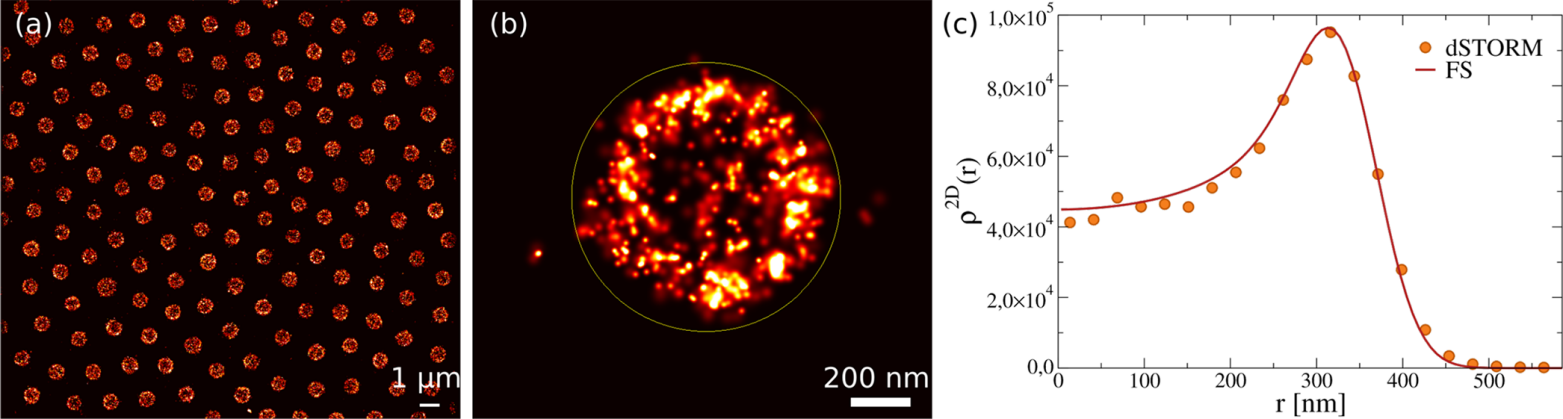
(a) dSTORM image of 1.5 mol % cross-linked, shell-labeled, swollen microgel particles at 25 °C. (b) Enlarged individual particle of the same sample. The rendering mode was set to individual localization precision, iso (Picasso). (c) dSTORM analysis of the microgel radial density profiles illustrating a measured 2D profile (symbols) (averaged over ∼100 different microgels) and the corresponding fit with the fuzzy-sphere model (line), as a function of *r*, the distance from the center of mass of the microgel.

To perform dSTORM experiments, microgels must be anchored to a surface, upon which they are irreversibly deposited by drying and resuspending them in water.^28^ In order to appropriately characterize the well-studied phenomenon of the VPT in pNIPAM microgels, we must thus ensure that this anchoring surface does not appreciably interfere with the microgel swelling and deswelling. To this aim, we use a hydrophilic surface, where the coverslip is treated with 3 M KOH, sonicated for 10 min, and then exposed for 10 min on a UV–ozone cleaner, obtaining surfaces with contact angles of less than 20°, as shown in Figure S1 of the Supporting Information (SI). For the imaging process we induce stochastic blinking of the fluorophores as described in Materials and Methodology. For each dSTORM image, we acquire 30000–60000 frames with an exposure time of 10–20 ms and proceed with image analysis using the open-source Picasso software,^38^ as detailed in the SI, following the methods of Conley and co-workers.^28,39^ Under these conditions we obtain an experimental resolution of around 30 nm in the plane (Figures S2–S5). In Figure 1 we show the typical experimental workflow for all microgel particles. We extract quantitative information from dSTORM using a customized MATLAB (MathWorks, USA) routine to determine the 2D fluorophore density profiles ρ^2D^(*r*) of the microgels as previously explained in ref (28). In order to describe the radially decaying density of the microgel under swollen conditions, the classic fuzzy-sphere model is used,^6^ adapted to the case where microgels solely contain fluorophores in their outer shell, with their denser core being “invisible” in dSTORM experiments, as discussed in Figure S6. The fuzzy-sphere model assumes that a microgel is made of a dense inner core of size *R* and a fuzzy shell of thickness 2σ_surf_. Both parameters can be obtained from a fit of the 2D density profiles taken from dSTORM, thus providing an estimate of the radius of the particle, *R*_tot_ = *R* + 2σ_surf_, projected on a plane. An example of the corresponding fit of the 2D density profile is reported in Figure 1c, showing good agreement with the dSTORM data.

**Figure 2:**
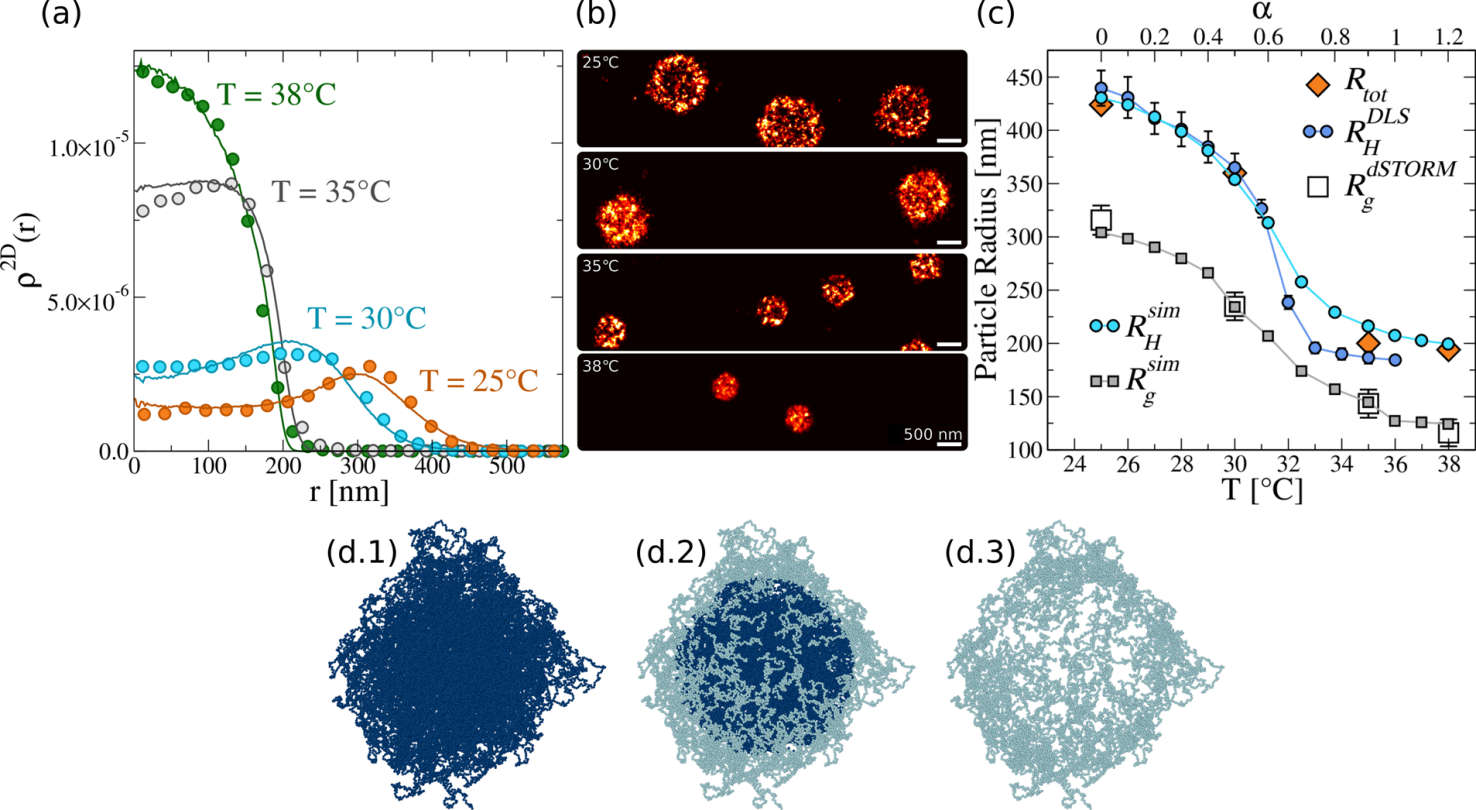
(a) Experimental 2D density profiles (symbols) for microgels at 25, 30, 35, and 38 °C and numerical 2D density profiles (solid lines) calculated with α_mm_ = 0.0, 0.5, 0.9, and 1.2, which best match the experimental profiles at the four studied temperatures. In particular, data at *T* = 25 °C are described without any noise on the fluorophore location (σ_sd_ = 0.0), while for *T* = 30 °C σ_sd_ = 0.3. At *T* = 38 °C, the microgels show a full collapse characterized by the complete absence of a hollow structure and are thus compared to the whole simulated microgel. Finally, for *T* = 35 °C we adopt a mixture of hollow (σ_sd_ = 0.4) and full microgels, as described in the text and Figure S9 for further details. In order to compare experimental and simulation density profiles, we normalize their area integral to 1. The simulated curves are reported on experimental units matching the respective 2D gyration radii, as explained in the text. (b) dSTORM images of individual microgels at 25, 30, 35, and 38 °C (top to bottom). For the complete captured region of interest (ROI), see Figure S3. (c) Comparison of dSTORM estimated radius *R*_tot_ as a function of temperature with the DLS *R*_H_^DLS^ and numerical *R*_H_^sim^ hydrodynamic radii. Also, 2D gyration radii from dSTORM *R*_g_^dSTORM^ and simulations *R*_g_^sim^ are reported. (d) Monomer-to-fluorophore conversion process: (d.1) initial full microgel, (d.2) monomer distinction according to their position either inside or outside the core–shell interface, and (d.3) final converted fluorophores.

The implementation of a temperature controller in the super-resolution setup allows us to observe the VPT of microgels *in situ*. We perform experiments at four different temperatures, 25, 30, 35, and 38 °C, covering the range where the transition occurs. The corresponding 2D density profiles are reported in Figure 2a, showing the typical deswelling behavior of the microgels across the VPT, also visible in the images of Figure 2b. From the fuzzy-sphere fits, we find that the microgel 2D radius *R*_tot_ (see Figure S6) decreases from *R*_tot_ ≈ 424 nm to *R*_tot_ ≈ 194 nm within the investigated temperature range. Moreover, the size of the microgel estimated by dSTORM is found to be in good agreement with the hydrodynamic radius *R*_H_^DLS^ measured by dynamic light scattering (DLS), as reported in Figure 2c.

To understand whether the anchoring to the hydrophilic surface may affect the swelling behavior of the microgels, we compare the experimental data with simulations of a realistic model of microgels,^40^ having the same nominal cross-linker concentration as the experimental sample.

We start for simplicity by showing results for simulations performed under bulk conditions: i.e., in the absence of a nearby surface. To appropriately model the deswelling of the microgel, we use an effective solvophobic potential, which controls the monomer–monomer attraction through an effective temperature α_mm_, as done in previous works^41^ and described in Materials and Methodology. In order to obtain a meaningful comparison between experiments and simulations, we mimic *in silico* the distribution of fluorophores detected by dSTORM. This is done by defining a smooth core–shell interface and converting to fluorophores all monomers belonging to the shell. The procedure ensures that the fluorophores are smoothly distributed across the interface as in the experiments, giving rise to a hollow profile as shown in Figure 2d. More details on the protocol employed to select the fluorophores can be found in Materials and Methodology and in the SI. We then calculate, as in experiments, the 2D radial density profiles of the simulated microgel across the VPT, only taking into account the external monomers and averaging over all three directions in order to improve statistics.

The resulting ρ^2D^(*r*) values are reported in Figure 2a, showing a nearly quantitative agreement with experiments for all the main features: the mass of the core and the extension of the corona as well as the presence of a peak (and its position) at low temperatures. Such a peak is a feature stemming from the combination of the 2D nature of the data and the fluorophore shell labeling, as shown in Figure S7. At temperatures below the VPT, we obtain a very good description of the density profiles with the addition of a small noise, that can be attributed to the finite optical resolution of dSTORM (see also Materials and Methodology and Figures S8 and S9). This is further illustrated in the fluorophore distributions with respect to their distance from the center of mass of the microgel, reported in Figure S10, which show that, as *T* increases, the employed fluorophore profiles move more and more toward the center of mass of the microgel. The situation becomes more complex at higher temperatures. First of all, we find that at *T* = 38 °C, the experimental profiles coincide with those of the full simulated microgel. Indeed, under these conditions microgels are fully collapsed and there is no longer evidence of a hollow structure from the images of Figure 2b. We interpret the latter as a consequence of several possible effects due to the 2-fold decrease in particle size above the VPT. First, the size of the microgel’s central region, where we expect a depleted fluorescence signal, is significantly reduced and shifted to a small area around the center of the microgel 2D projection. For small *r* values, however, the radially averaged dSTORM signal is very noisy due to a small number of fluorophore localizations. Moreover, the depth of field and the microgel size are comparable, which may lead to subtle changes in imaging conditions upon shrinkage.^42^ In addition, we should consider the possibility that shrinkage leads to a slight redistribution of the dye-labeled polymer strands across the volume, blurring the hole signal. Furthermore, the refractive index of the microgels increases to a degree where we cannot entirely neglect the scattering and attenuation of the exciting light beam. Deciphering the various contributions to the disappearance of the apparent hole is beyond the scope of this paper but will be addressed in future work.

Interestingly, at *T* = 35 °C, just above the VPT, we find the presence in the samples of fluctuations within the different microgel reconstructions, so that some of them clearly display a hole, while some others do not. This variability could be attributed to a different illumination or inclination that can vary somewhat from one measurement to another, as well as to the large experimental uncertainty near *r* = 0. We consider this experimental observation in our modeling as discussed in detail in Figure S9, which leads us to separately account for the profiles of the two microgel populations and appropriately mix them in the right proportion in order to obtain the 35 °C profile shown in Figure 2a.

It is evident from Figure 2a that the decay at large distances is extremely well captured at all temperatures, signaling that the simulations are able to fully grasp the amount of shrinking observed in experiments. To this aim, we note that a previous work^41^ had established a mapping between temperature and solvophobic parameter α_mm_ by comparing numerical form factors with experimental factors of PNIPAM microgels obtained by small-angle X-ray scattering. Using dSTORM, we now confirm similar values of α_mm_ in the studied temperature range. Small deviations occur probably due to differences in the synthesis process. To convert numerical into experimental units, we impose that the 2D radius of gyration of the microgel at α_mm_ = 0.0, i.e. in the absence of any monomer–monomer attraction, has to be equal to the experimental value obtained at the lowest temperature, *T* = 25 °C.^43^ With this procedure, we find that the numerical unit length σ, corresponding to the size of a monomer bead in the model, corresponds to ∼9 nm in real units. Such a conversion is then used at all temperatures and throughout the paper. The resulting numerical and experimental 2D gyration radii, *R*_g_^sim^ and *R*_g_^dSTORM^ respectively, are found to be in good agreement at all temperatures, as reported in Figure 2c. In addition, we also calculate the hydrodynamic radius *R*_H_^sim^ numerically using the Zeno algorithm,^44^ which was recently validated for microgels,^45,46^ and again we find an overall satisfactory agreement between experiments and simulations. The slight discrepancy observed at high temperatures with respect to the DLS data may likely be due to the fact that the dSTORM buffer solution is different from that used in DLS measurements. Indeed, for the same buffer conditions as in dSTORM, DLS measurements of *R*_H_ are not possible at high temperatures, because of the onset of microgel aggregation.

Overall, the agreement of numerical simulations in the absence of a surface with dSTORM data suggests that the anchoring of microgels in experiments has a negligible effect on the particle structure throughout the volume phase transition. To confirm that this is the case, we also perform simulations in the presence of a nearby hydrophilic surface. Since the interactions between monomers and wall is mainly repulsive, the surface does not affect the results at any temperature, because the microgel always remains relatively far from the surface. We then mimic the experimental procedure by anchoring a small fraction of microgel monomers on the surface, as detailed in Materials and Methodology. By adding the surface, we first need to quantify the effect of the number of anchoring sites, which we have assessed in Figure S11, by a direct comparison to experiments. From this, we get a rough estimate of the fraction of monomers bonded to the surface that is found to be smaller than 0.1%. Using such a value, we find that the wall-anchored density profiles are equivalent to those in the bulk as shown in Figure S12, confirming that the microgel structure across the VPT remains unperturbed when anchored to a hydrophilic surface. Altogether, these results validate the adopted experimental procedure, indicating that they can clearly detect the changes in the internal structure of the particles at different temperatures. We expect that dSTORM will be very useful to study different systems, such as copolymer microgels with more complex internal architectures, where different temperature behaviors of the forming polymers are at play.^13,47,48^

### Changing the Surface Affinity: the VPT of Microgels Close to a Hydrophobic Surface

**Figure 3:**
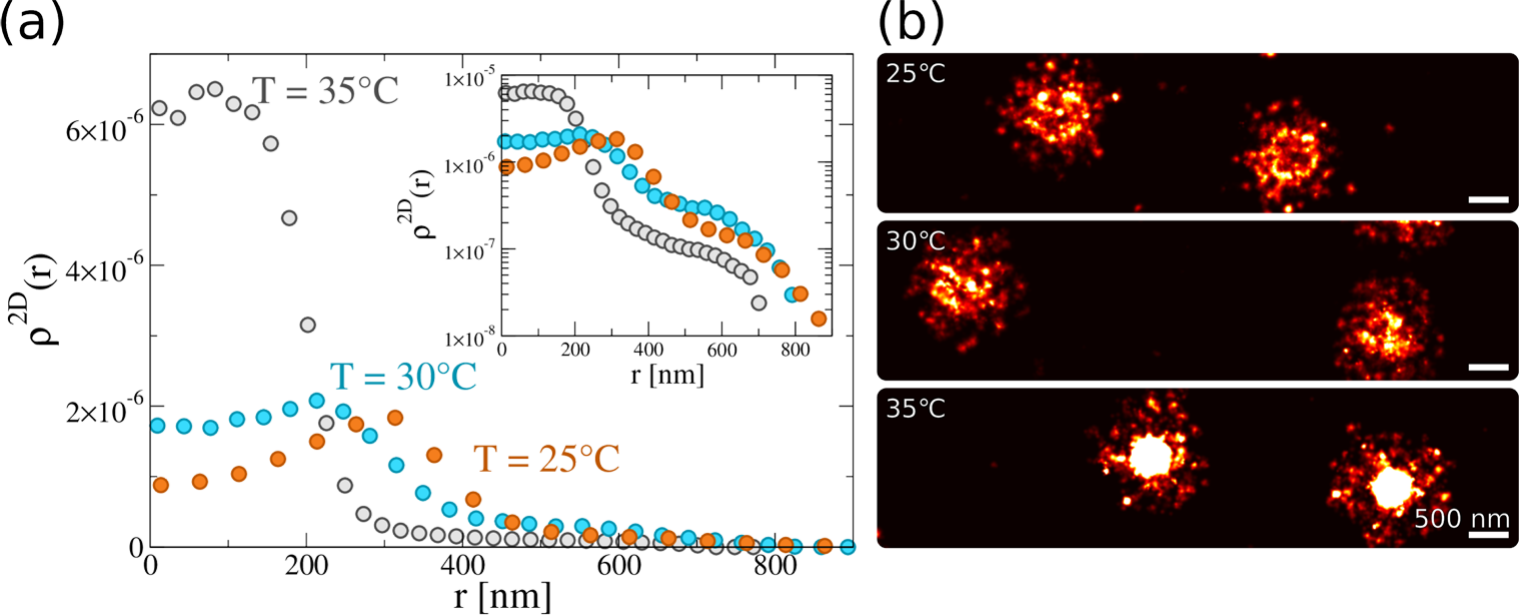
(a) Experimental 2D density profiles for microgels at 25, 30, and 35 °C on a surface exposed to HMDS. Inset: same density profiles with the *y* axis in logarithmic scale to better visualize the tails of the profiles. (b) dSTORM images of individual microgels at 25, 30, and 35 °C (top to bottom). Here the brightness of the image at 35 °C is increased to make the anchoring parts clearly visible for the reader. For the complete captured ROI, see Figure S4.

We now discuss the case of microgels close to a hydrophobic surface. To realize this situation, the coverslips were first cleaned with 3 M KOH and then exposed overnight to 0.1 mL of hexamethyldisilazane (HMDS). Contact angle measurements are shown in Figure S1, reporting contact angles larger than 80°. The resulting 2D density profiles are reported in Figure 3a, still showing a clear deswelling of the particles with temperature, accompanied however by the presence of a long tail at large distances, which persists even under the more collapsed conditions. To visualize this behavior, dSTORM images at three studied temperatures are shown in Figure 3b, clearly indicating that the microgels adopt a core–shell-like arrangement, since the external shell tends to maximize the contact with the surface. This behavior is in agreement with previous super-resolution experiments,^35^ which were performed only at low temperatures. The present results demonstrate that the tail is maintained even at high temperatures, denoting a large affinity of the microgel to the surface. It is also interesting to compare these results with observations at liquid–liquid interfaces, where microgels adopt the so-called “fried egg” configuration, confirmed by a large number of experiments (mainly through atomic force microscopy, after deposition onto a surface)^49^ as well as numerical simulations.^50^ Interestingly, in the case of a liquid–liquid interface, temperature effects on the microgel conformation are not very pronounced^51^ because of the dominant role of the interfacial tension, as also recently confirmed by direct investigation through *in situ* neutron reflectometry.^52^ Hence, it is worth examining in more detail the role played by temperature in the present work, where it seems that a competition between hydrophobic interactions, monomer–monomer vs monomer–surface, is at work.

**Figure 4:**
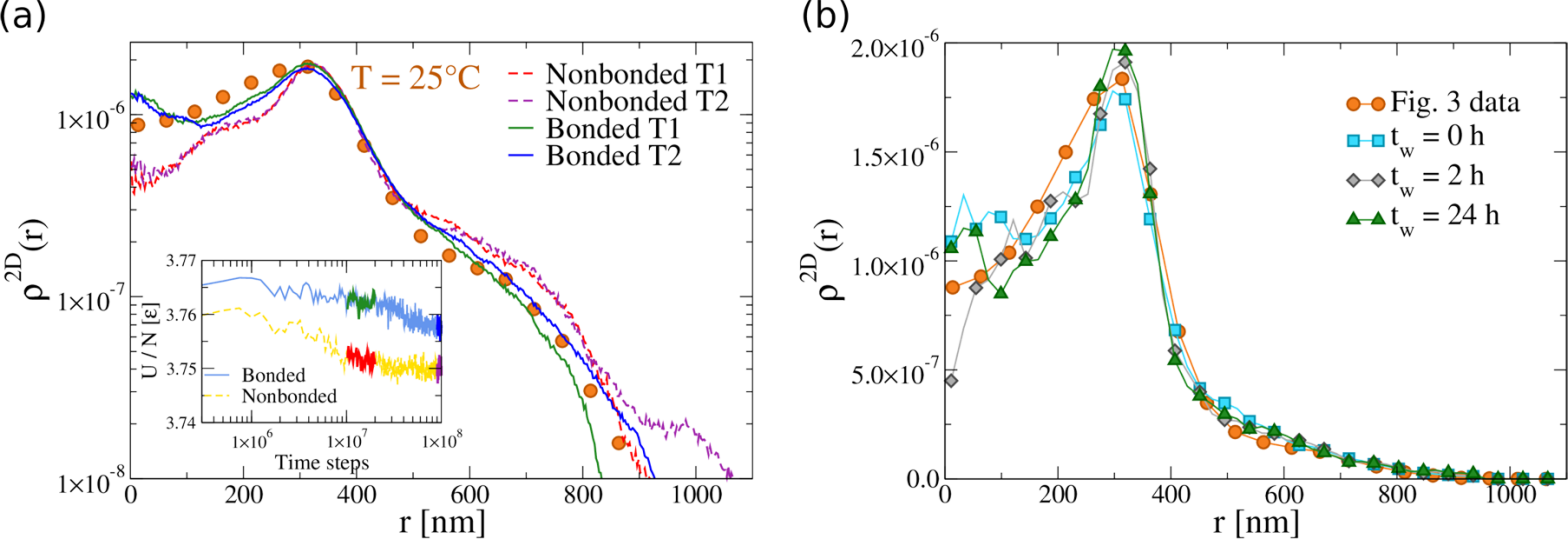
(a) Experimental 2D density profiles (symbols) for microgels at 25 °C and corresponding numerical ones obtained with α_mm_ = 0.0 and α_ms_ = 0.9 at different times for unbonded (dashed lines) and bonded microgels (solid lines). The time intervals are displayed in the inset. Inset: evolution of the potential energy per particle of the bonded and unbonded microgels versus time in numerical simulations. The highlighted regions indicate the time intervals where the correspondingly colored curves have been calculated in the main panel. (b) Additional measurements for 2D density profiles at 25 °C and waiting times *t*_w_ = 0, 2, and 24 h in comparison to the data reported in Figure 3a and elsewhere in the paper.

To this aim, we resort to numerical simulations and we model the interactions between monomer and surface with the same potential shape as for the monomer–monomer case. In this case, the parameter α_ms_ controls the affinity between monomers and wall particles. While in the case of a hydrophilic surface we set α_ms_ = 0, we now tune α_ms_ > 0 to model the hydrophobicity of the surface, as detailed in Materials and Methodology. We separately consider in the following both the case of a free microgel next to the attractive surface (unbonded), which spontaneously sticks to it, and that of microgel anchored to the surface (bonded). After tuning α_ms_ as shown in Figure S13, we find that the best agreement between experiments and simulations is obtained for α_ms_ = 0.9, i.e. a relatively large value of the attraction strength, confirming the rather high hydrophobic character of the HMDS surface. We compare the resulting numerical and experimental 2D density profiles at *T* = 25 °C in Figure 4a and note that the system takes a very long time to reach equilibrium. This is illustrated in the inset, reporting the energy per particle versus time, which is found to slowly decrease, denoting a long aging regime, for both bonded and unbonded microgels. During this time, also the density profile of the microgel slowly evolves, accumulating more and more monomers at large distances from the center of mass. This indicates that, for the chosen conditions, the hydrophobic attraction of the monomers to the wall is dominant and pushes the microgel to extend farther and farther on the surface. Interestingly, from the analysis at different values of α_ms_, we found that only for α_ms_ ≳ 0.7 does the microgel adheres to the surface, otherwise it tends to remain in a spherical (almost unperturbed) condition. However, as soon as the microgel sticks, slow rearrangements of the monomers on the surface take place, giving rise to this non-negligible long-time evolution. These results show that, on increasing time, the outer shell progressively extends more than in the experiments, thus overestimating the tail of the density profiles in the case of an unbonded microgel. This is also reflected in the short-distance behavior of the numerical ρ^2D^(*r*), whose height is lower than the height observed with dSTORM. Instead, looking at the numerical data for a bonded microgel, also reported in Figure 4a, we find that the experimental data are much better captured and, in particular, at long simulation times the tail of the distribution roughly extends the same amount as in experiments. Hence, differently from the case of a hydrophilic surface, here a small degree of anchoring does affect the overall conformation of a microgel due to the strong binding to the surface and the underlying connectivity of the network. We thus conclude that we cannot neglect the presence of anchoring in the simulations for a more quantitative description of super-resolution data close to a hydrophobic surface.

To verify whether this holds at all *T*, we extend the comparison to higher temperatures to tackle the interesting case of a collapsing microgel close to a hydrophobic surface. To this aim, several details need to be taken into account. The first point to address is aging, which is very pronounced in the simulations, due to the fact that, at high *T*, there is a competition between the monomer–monomer attraction, modeled by α_mm_ = 0.9, and the monomer–surface attraction, modeled with α_ms_ = 0.9, as determined at *T* = 25 °C. In particular, we find that the monomer–monomer attraction, being augmented by the large number of nearby monomers and the overall connectivity, will eventually dominate when the two α parameters are the same, so that the microgel will tend to collapse further, decreasing the extent of the large distance tail. It is now important to assess the role of aging on the experimental results. To this aim, we performed additional measurements for microgels on the hydrophobic surface at different waiting times *t*_w_ for *T* = 25 °C. We define *t*_w_ = 0 as the time when we started the dSTORM measurements. When comparing the density profiles of measurements done at *t*_w_ = 0, 2, and 24 h, reported in Figure 4b, we detect no significant differences in the curves. Indeed, the time between sample preparation and data acquisition, which is roughly of the order of 1 h, seems to be long enough for the system to reach equilibrium, so that we can neglect aging effects in the experimentally measured time window. These results should thus be compared with numerical results at very long times.

Next, we consider the fact that at high *T* the density profiles may also depend on the way the microgel is anchored on the surface. To address this problem, we consider the anchoring process of a swollen microgel (see Materials and Methodology) both onto a hydrophilic and onto a hydrophobic surface. This yields different bonding patterns: when the anchoring process is done on a hydrophobic surface the bonds tend to be made for monomers located farther away from its plane projected center of mass. We then calculate ρ^2D^(*r*) above the VPT (see Figure S15) and find that the presence of a hydrophobic surface allows for the formation of bonds over a more extended region compared to the hydrophilic surface, which results in a larger tail of the density profile. This is in closer agreement with experiments, because it more realistically mimics the way that the anchoring is made.

**Figure 5:**
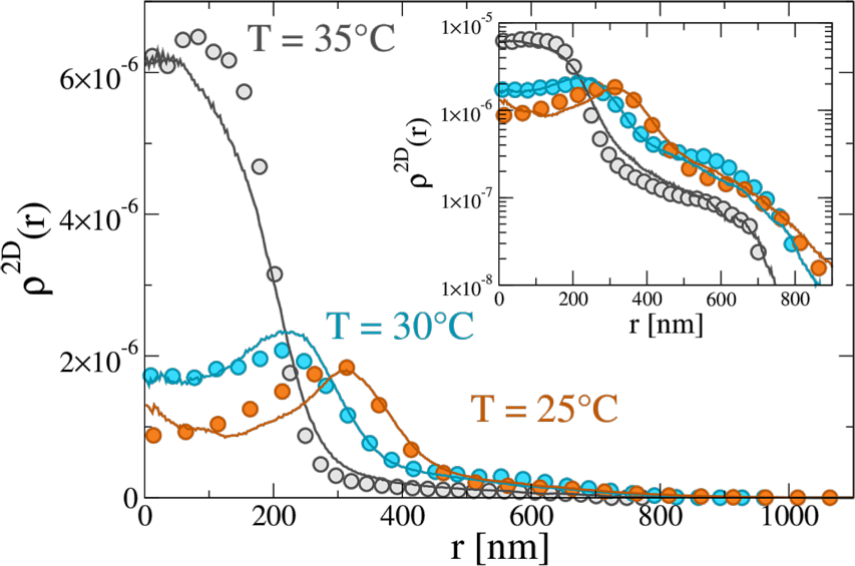
Experimental 2D density profiles (symbols) and numerical 2D density profiles (solid lines) calculated with α_ms_ = 0.9 and α_mm_ = 0.0, 0.5, 0.9. Data for *T* = 25 °C are described without any noise on the fluorophore location (σ_sd_ = 0.0), while for *T* = 30 °C σ_sd_ = 0.3, in analogy with the hydrophilic case. Instead, data for *T* = 35 °C show the absence of a hollow structure and are thus compared to the whole simulated microgel. Inset: *y* axis in logarithmic scale.

Having established the optimal ways of comparing experiments and simulations, we are now able to finally report the comparison of experimental and numerical density profiles at all investigated temperatures close to the hydrophobic surface in Figure 5. Below the VPT temperature (*T* = 25 and 30 °C) the profiles still show the presence of a peak, indicative of the fluorophore outer-shell distribution, that we keep exactly identical with that used in the presence of the hydrophilic surface (see Figure S10). However, at the highest studied *T* = 35 °C the experimental profile shows a monotonic decrease at low distances, being characterized by the absence of a hollow structure, similarly to what observed at high temperatures for the case of a hydrophilic surface. Indeed, we find that the comparison with any fluorophore distribution is not able to reproduce the experimental data (see Figure S15), but instead we consider the full microgel profile and obtain a very satisfactory agreement. This happens at a slightly lower temperature with respect to the hydrophilic case, probably due to the presence of the attractive surface, which effectively favors an anticipated collapse of the microgel. Overall, Figure 5 shows that simulations, taking appropriately into account the subtleties of anchoring and fluorophore detection, can capture the experimental data at all temperatures also in the case of a hydrophobic surface.

**Figure 6:**
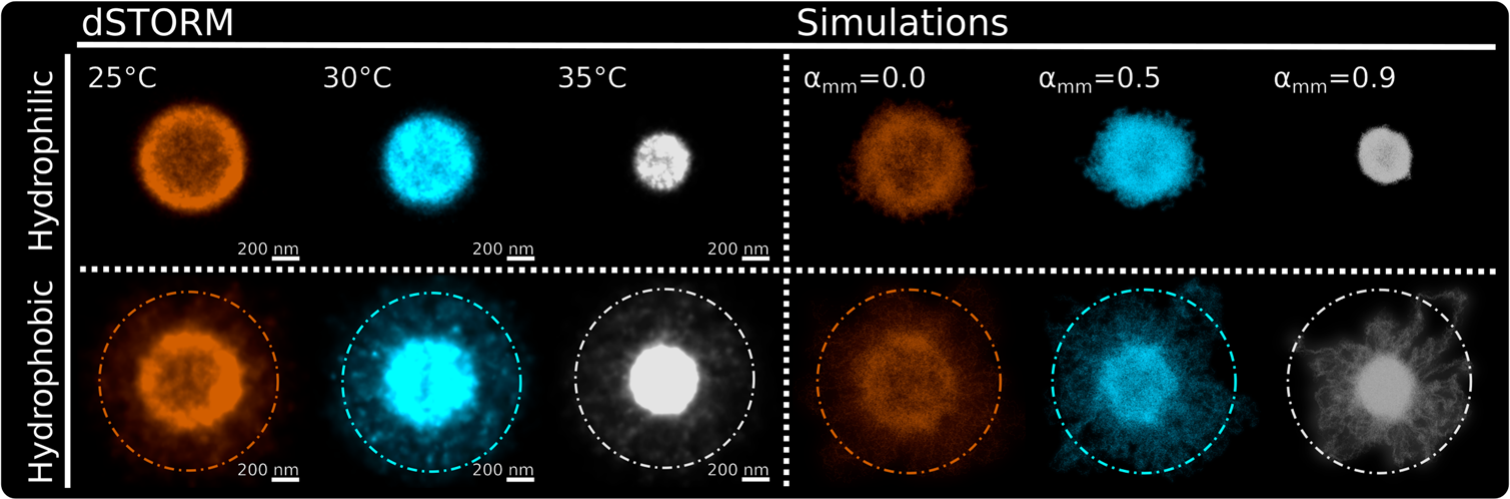
Averaged images of individual microgels for increasing temperatures (25, 30, and 35 °C) on a hydrophilic surface (top panels; dSTORM left, simulations right) and on a hydrophobic surface (bottom panels; dSTORM left, simulations right). The brightness for the images on a hydrophobic surface is increased to make the anchoring parts clearly visible for the reader.

The quantitative comparison between experiments and simulations can be summarized in Figure 6. This reports the average images, recorded by dSTORM, of individual microgels at three studied temperatures and close to the two different surfaces. While for the hydrophilic case the typical spherical pattern is retained, for the hydrophobic surface the shell around the core persists at all temperatures, in very good agreement between experiments and simulations. In addition, it is clear from the direct comparison of the images for 35 °C for the two examined cases that the microgel internal structure is much more compact (without a perceptible hole in the center) for the hydrophobic conditions, in agreement with the employed numerical description.

## Conclusions

In this paper we performed a detailed investigation of the volume phase transition of standard pNIPAM microgels by dSTORM measurements combined with coarse-grained numerical simulations of realistic microgels. The experiments were carried out by anchoring the microgels to two different substrates: a fully hydrophilic and a largely hydrophobic surface.

In the former case, we found that the presence of the nearby wall does not significantly affect the conformations of the microgels, which always remain spherical at all investigated temperatures. It was thus possible to monitor the occurrence of the VPT and to quantitatively compare with simulations of isolated microgels in the bulk and on hydrophilic surfaces. The comparison was performed by adopting a procedure where only a fraction of monomers, those emulating the fluorophores, were taken into account in the calculation at low temperatures. Instead, with the increasing collapse of the microgel, the hollow core progressively becomes detectable, so that the profile is well captured by that of the full microgel. The favorable agreement of experiments and simulations, on one hand, reinforces the numerical model, previously shown to be able to describe the evolution of microgels form factors across the VPT^41^ and now validated also in direct space, and on the other hand, establishes the use of the temperature-controlled dSTORM approach employed in this work for the nanoscale resolution of the internal structure of microgels. Indeed, despite the technique requiring anchoring onto a surface, the successful comparison with simulations indicates that such anchoring can be safely neglected in the description, because it does not affect the overall swelling–deswelling process. To this aim we showed that the dSTORM measurements are quantitatively captured also by simulations performed in the absence of a surface.

Having validated the combination of our methods, we then moved on to examine the influence of the surface on the VPT when interactions between monomers and substrate are highly attractive. For the latter case, we are not aware of any previous detailed characterization of the microgel conformation attached to a hydrophobic surface as a function of temperature. While at low temperatures, in agreement with previous works,^35,36^ the preferential attraction to the surface induces the microgel to spread and deform, rather similarly to the case of liquid–liquid interfaces^50,53^ and thus adopting a core–shell-like structure, the previously unexplored high-temperature regime reveals an interesting behavior. This is due to the emerging competition between microgel deswelling, controlled by the decrease in the solvent–monomer affinity, and the adhesion to the surface, modulated by the hydrophobic coating of the substrate. Thanks to the help of simulations, where these two parameters can be varied more easily than in experiments, a delicate interplay between the two mechanisms arises, giving rise to a subtle aging behavior in the simulations. However, in experiments, the typical preparation time is about 1 h, which allows for a full equilibration of the system, so that we can safely neglect aging effects in the measurements. In the simulations, we instead find that a very long equilibration takes place, which does not change the overall shape of the density profiles but may affect the tail of the profiles. To determine the long-term behavior of such tails, it turns out to be crucial to appropriately consider the presence of the anchoring of the microgels to the substrate. In particular, in the absence of anchoring, our simulated microgels would continue to spread even further under the studied conditions, a process that is inhibited by the presence of permanent bonds with the surface which, combined with the network structure of the particles, limits the growth of the tail at long times, in good qualitative agreement with experiments. Importantly, comparing the present results for the hydrophobic surface with those obtained at liquid–liquid interfaces, we note that the use of a solid substrate enables a more effective exploitation of temperature effects on the final microgel configuration. Indeed, we find a rather exotic conformation made of a collapsed core plus an extended shell due to the interplay between the two tunable and competing hydrophobic strengths. Such a conformation is not easily observed at liquid–liquid interfaces, because in that case the interfacial tension is always dominant with respect to temperature.

In future works it will be interesting to extend the present results to the investigation of different surfaces, varying the hydrophobic affinity of the microgel^32^ as well as varying the cross-linker concentration. While here we focused on rather soft microgels with a low amount of cross-linkers, we expect that a variation of the particle softness^54^ will produce additional interesting features. In particular, the use of ultralow-cross-linked microgels may be especially worth exploring, due to their intrinsic difference with respect to standard microgels.^52,55^ In addition, a careful comparison between microgel conformations at a liquid–solid vs a liquid–liquid interface, building on that performed by AFM measurements,^55,70^ would be desirable to fully unveil the conformational differences of the particles with nanoscopic resolution.

The present study strongly suggests that dSTORM can also be used to investigate the VPT behavior of microgels with a complex inner structure, including copolymerized microgels with different responsivities with respect to temperature or pH or different internal architectures: e.g., interpenetrated network microgels. To this aim, it would be desirable to first extend the present analysis to different labeling over the whole microgel and not only on the surface, as previously done in ref (28), to be able to detect variations throughout the particles, even in the core. In addition, a full 3D imaging should be implemented so that the full density profiles could be directly compared to simulations or to experimental form factors, although this is not crucial in the presence of microgel deformation onto a surface, such as that studied in this work.

Finally, an additional step foward will be to move from simple to compartmentalized microgels^30^ useful for segregating reactive components and coordinating chemical reactions or to nanocomplexes where these are decorated with other objects, such as nanoparticles, e.g. for enhancing plasmonic or optical properties,^56^ or biomolecules, e.g. for delivery purposes.^57−59^ In these cases, the advanced super-resolution approach developed in the present work will enable the visualization of the full temperature behavior *in situ*, which is crucial to control adsorption and release of these molecules with high potential for their fundamental mechanisms occurring at the nanoscale and to improve their applications in different fields.

## Materials and Methodology

### Microgel Synthesis

We synthesized pNIPAM microgels using the free radical precipitation polymerization method as previously described by Conley et al.^28^*N*-Isopropylacrylamide (Acros Organics, 99%; NIPAM) is the monomeric unit which is recrystallized in hexane before use and *N*,*N*-methylene bis(acrylamide) (Sigma-Aldrich, 99%; BIS, is the cross-linker. In addition, *N*-(3-aminopropyl) methacrylamide hydrochloride (Polysciences; APMA) is added as a comonomer to incorporate free amine groups into the microgel network. The primary amines are used to fluorescently label the microgels by reacting with the succinimidyl ester groups of the dye AlexaFluor 647. 2,2-Azobis(2-methylpropionamidine) dihydrochloride (Sigma-Aldrich, 98%; AAPH) is used to initiate the polymerization. First, in a three-neck round-bottom flask, NIPAM (1.430 g) and BIS (0.029 g) are dissolved in 85 g of H_2_O. The reaction mixture is purged with nitrogen for 30 min before the temperature is raised to 70 °C. Next, 0.0365 g of AAPH, previously dissolved in 5 g of H_2_O, is added to the reaction mixture. Five minutes after the initiator is added and the solution has started to turn white, 0.0198 g of APMA dissolved in 10 g of H_2_O is introduced to the reaction mixture using a syringe pump at an addition rate of 0.5 mL/min. The reaction mixture is kept at 70 °C for 4 h before cooling down rapidly in an ice bath. Following this protocol, inhomogeneous core–shell microgels are formed with APMA comonomer only being present on the shell of the microgel network. A purification step follows to remove all unreacted monomers in the solution by centrifugation (three to four times). We label the microgels using an excess amount of fluorescent dye Alexa 647 and leave them in an oscillation plate for 1 h before another purification step to remove all unreacted dye.

### Super-Resolution Microscopy

To perform dSTORM experiments, we use a Nikon TiEclipse inverted microscope with an EMCCD camera (Andor iXon Ultra 897) and total internal reflection fluorescence (TIRF) arm to achieve highly inclined illumination and limit the fluorescence background noise. We use a continuous-wave red laser, coherent Genesis MX-STM with 1000 mW output power at 639 nm, providing a single-mode TEM00 Gaussian beam, horizontally polarized. The high-power red laser enables fluorophores in their excited state through intersystem crossing to occupy the triplet state where they get trapped. A second laser (Toptica iBeam Smart), with 120 mW output at 405 nm, vertically polarized, is used to tune the blinking density. Both lasers are coupled into a single-mode fiber (S405XP, Thorlabs) into the TIRF arm. The light is focused on the back aperture of a high-numerical aperture and magnification objective (NA 1.49 and 100× magnification). With an extra zoom lens placed before the camera, the final pixel size is 110 nm. A dichroic filter with a wavelength of 700 nm and bandwidth of 50 nm is placed in the detection pathway (ET700/50, Chroma).

First, we set the appropriate buffer conditions at 50 mM cysteamine, and pH is adjusted to 8. Second, we illuminate the sample at a wavelength of λ = 639 nm using a high laser power (3.4 kW/cm^2^) to bring the fluorophores to a metastable dark state. We employ a second laser at 405 nm to induce stochastic spare fluorescent light emission and control the blinking density by adjusting the laser power. We acquire 30000–60000 frames with an exposure time of 10–20 ms. Using Picasso software, for each frame we localize individual blinking events that we then fit with a 2D Gaussian profile using the maximum likelihood estimation method (MLE). Following this procedure, we generate a database that contains *x*–*y* positions, intensity, and localization precision (*x*, *y*). To reconstruct the entire super-resolution image, we filter the list of localization and only retain localizations above a certain photon count threshold, with low ellipticity (<0.2) and with a sufficient localization precision in *x* and *y* (<0.2 pixel). We correct the reconstructed image’s drift using a redundant cross-correlation algorithm and then render the image using the individual localization precision (iso) mode unless otherwise stated.

For the aging measurements, we took into account that the number of localizations may vary with time, due to the fact that the buffer conditions change over a long imaging time.^60^ In order to compare among the different waiting times, we then consider only the first 10000 blinking frames of the measurement, corroborating that the number of localizations per microgel (about 3000) were comparable for all cases.

### Light Scattering Measurements

DLS measurements are performed using a commercial LS Spectrometer, a 2D-DLS Pseudo cross correlation setup (LS Instruments AG, Switzerland). We increase the temperature from 25 to 35 °C with Δ*T* = 1 °C as the step size. Laser light with a wavelength of 660 nm was used to perform the experiments at scattering angles of 40–80° with 5° step size. Three measurements of 40 s were obtained at every angle. The diffusion coefficients *D* and hydrodynamic radii *R*_H_^DLS^ were extracted from a multiangle analysis of the first-order cumulant fit.

### Numerical Methods

#### Microgel Modeling

We simulate the microgel assembly following the methods described in refs (40 and 41). The assembly method is a two-step process: first, (i) bi- and tetravalent patchy particles self-assemble inside a spherical cavity, forming a fully bonded disordered network; then, (ii) the topology of the network gets fixed by replacing the patchy interactions with permanent bonds. For the fixed topology, particles composing the microgel, which we will also refer to as monomers, interact with the Kremer–Grest bead–spring model:^61^ i.e. monomer overlap is avoided with the repulsive Weeks–Chandler–Andersen (WCA) potential^62^
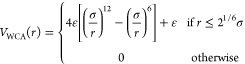
1while permanent bonding between monomers is ensured by adding the Finite-Extensible-Nonlinear-Elastic (FENE) potential for bonded pairs^63^
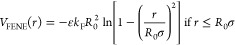
2where σ is the monomer diameter, ε is the energy scale, *k*_F_ = 15 is the dimensionless spring constant, and *R*_0_ = 1.5 is the maximum bond extension. The volume phase transition of the microgels is reproduced by adding an attractive solvophobic interaction *V*_α_mm__(*r*) among monomers.^64,65^ The attraction strength is controlled by the parameter α_mm_, which encodes the monomer–monomer effective attraction, modeling implicitly the reduction of monomer–solvent affinity when increasing the temperature
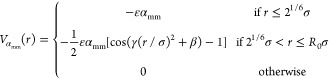
3with γ = π(2.25 – 2^1/3^)^−1^ and β = 2π – 2.25γ. When α_mm_ = 0 no implicit-solvent attraction is added between monomers, reproducing good solvent conditions and a swollen microgel; instead, monomer attraction increases by increasing α_mm_, thus shrinking the microgel size and mimicking the worsening of the implicit solvent.

#### Solid Surface Modeling and Monomer Anchoring

To reproduce the behavior of the microgel close to a surface, we model the latter as two layers of wall particles that are initially located on a compact square lattice of size σ at the bottom of the simulation box; the separation between layers is 0.7σ. To avoid crystallization of the monomers close to the surface, the wall particles are randomly displaced from the lattice sites, including the direction perpendicular to the plane, following a Gaussian distribution with standard deviation σ_sd_ = 0.2. The obtained layers are then subsequently fixed throughout the whole simulation runs.

Microgel monomers interact with wall particles via the WCA potential (eq 1) and the *V*_α_ms__ potential, which is identical with eq 3, but this time replacing the monomer–monomer attraction α_mm_ with the monomer–surface attraction, α_ms_, now encoding the surface hydrophobicity.

**Figure 7:**
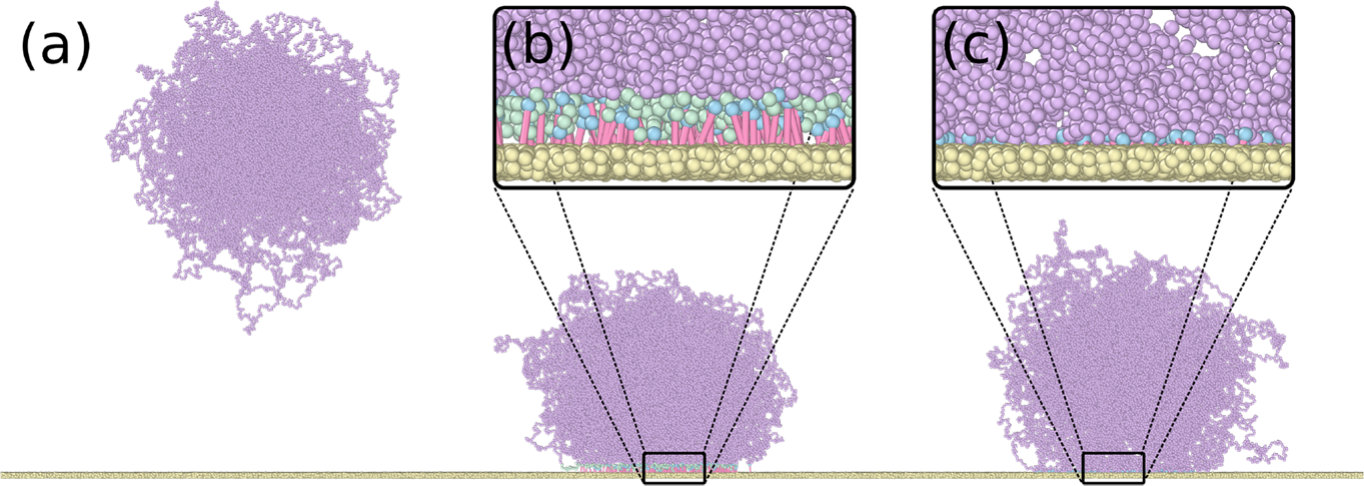
Bonding of the microgel to the hydrophilic surface (a) The microgel in the bulk is pushed toward the wall. (b) When in contact, a list is made with all monomers at a distance to the wall smaller than *dz* (monomers in green), from which some are randomly chosen (blue) and bonded to their closest wall-particle (pink bond). (c) The microgel is left to relax.

To mimic experimental conditions where the microgel is physically anchored to the wall, we also consider the case where permanent bonds between a few monomers and the surface particles are formed. This is obtained by the following procedure (illustrated in Figure 7 for the hydrophilic scenario α_ms_ = 0): (i) a swollen microgel (equilibrated in bulk at α_mm_ = 0) is pushed toward the wall; (ii) when it comes in contact with the surface, monomers with distance less than *dz* = 1.5σ from the upper layer of the wall are considered, and among them; (iii) *b* monomers are randomly chosen and anchored to their closest wall-particle via the harmonic potential *V*(*r*) = *K*(*r* – *r*_0_)^2^ with *K* = 15 and *r*_0_ = 2^1/6^σ; finally (iv) the microgel is left to relax to its equilibrium state. The procedure is then repeated for different surface α_ms_ conditions. We did it for both the hydrophilic α_ms_ = 0 and hydrophobic α_ms_ = 0.9 conditions, yielding different bonding patterns. Density profiles calculated with microgels anchored to a hydrophilic surface are comparable to experiments in all cases, except for the measurements at *T* = 35 °C close to a hydrophobic surface, where we found that the extension of the tail is better captured by simulations of microgels initially anchored to a hydrophobic surface (see Figure S15). Another important parameter to take into account is the number of bonds *b* that we should consider in the simulations. As mentioned in Results and shown in Figure S11, for the hydrophilic surface we tried different values of *b* and found that *b* = 25 is the most similar to experimental data. For the hydrophobic case, we expect in experiments a much larger number of bonds due to the additional attraction to the surface in the anchoring procedure. For this reason, we performed all simulations (that take much longer, also due to the long aging regime) with a fixed value *b* = 200, roughly 1 order of magnitude difference with respect to the hydrophilic case. This value was then found to be in rather good agreement with experiments in terms of the tail of the density profiles.

#### Fluorophore Emulation

Super-resolution experiments only detect the presence of fluorophores labeling the microgels. We thus need a way to go from the full monomer representation of the microgel to the case of only counting fluorophores in the calculation of the density profiles to mimic the partial labeling performed in the experimental synthesis. In our simulation this is equivalent to choosing a particular set of monomers that are considered to be the fluorophores. Since microgels are synthesized in such a way that these are mostly located in the surface, the monomer-to-fluorophore conversion process must take this into account. To this aim, we perform the fluorophore selection with the following procedure, illustrated in Figure 2d: we allocate monomers to the *fluorophore list* according to their distance *r* from the microgel center of mass (CM) in a single equilibrated configuration by testing if *r* > ⟨*R*_*g*_⟩·*P*(σ_sd_, μ = 1), where *P*(σ_sd_, μ = 1) is a random number taken from a Gaussian distribution of mean μ = 1 and standard deviation σ_sd_. The addition of such a Gaussian noise softens the definition of the interface and takes into account averaging over different equilibrium configurations as well as the fact that there could be mislocalization or loss of resolution in the experiments. Indeed, we find that σ_sd_ increases with temperature, due to the fact that it is more difficult to resolve the core–shell interface and the position of the fluorophores once the microgel collapses, as shown in Figure S8. We find that σ_sd_ = 0.0 and 0.3 for 25 and 30 °C, respectively. However, at high temperatures, when the microgel approaches a full collapse, the fluorophore description loses its validity. In particular, at 38 (35) °C for the hydrophilic (hydrophobic) case, respectively, as discussed in the main text, the density profiles do not show a peak in the outer shell, and we resort to considering all the monomers to calculate the numerical 2D density profiles. Instead, for 35 °C and a hydrophilic surface a mixture of the two approaches is needed, because fluctuations from microgel to microgel are large and we have almost an equal population of fully resolved and hollow profiles. We thus average them following experimental proportions, using σ_sd_ = 0.4 for the fluorophore distribution.

#### Simulation Parameters

Molecular dynamics (MD) simulations of three independent realizations of microgels with different topologies were performed using LAMMPS.^66^ Microgels were assembled using the oxDNA package^67^ starting with *N* = 42000 patchy particles, of which 1.5% are tetravalent (cross-linkers) to match experimental conditions. It is important to note that the adopted *N* yields the size of a monomer to be around 9 nm, as described in the text. This value is slightly larger than the estimated Kuhn length for PNIPAM,^68^ but we previously showed^41^ that, upon increasing the number of monomers, the bead size approaches the correct value without qualitatively affecting the results. The wall is made of two layers of 90000 wall-particles each. Hence, each system has about 222000 particles in total.

We used a Langevin thermostat with reduced temperature T* = *k*_B_*T*/ε = 1, particle mass *m* = 1, and integration time . Wall-particles are kept fixed by not including them in the integration scheme. The length of the simulations changes for the different scenarios; we monitored the energy as to see when the system had thermalized and then ran additional steps from where the density profiles were calculated. Simulations in the bulk and on a hydrophilic surface take around 1 × 10^6^ steps to equilibrate. After this, we ran additional 15 × 10^6^ steps. Density profiles are calculated from configurations in the last 5 × 10^6^ steps of the simulation. Instead, the evolution of microgels placed close to a hydrophobic surface is very slow. Hence, in this case we performed 100 × 10^6^ steps. We calculated the profiles at different waiting times and found that their shape did not show significant changes (except at very large distances), similar to what is reported in Figure 4.

The radial density profiles of individual equilibrated microgels were calculated as
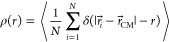
4where ⟨·⟩ is the average over several configurations. When calculating the 2D profiles, *r⃗* and *r⃗*_CM_ do not include the component perpendicular to the surface. Observables were averaged over three independent microgel configurations in order to avoid singular topology characteristics that may occur. Images of numerical microgels were created with OVITO.^69^

## Data Availability

The data shown in the figures and some of the underlying raw data are available on the online repository Zenodo (https://doi.org/10.5281/zenodo.7515402).

## Supporting Information Available

Additional details on the sample preparation for dSTORM measurements, the image analysis, and the density profiles fit using the fuzzy-sphere model for microgels on the hydrophilic surface and additional simulation details regarding the 2D projected density profiles, the estimation of the fluorophore distribution, the comparison between bulk and hydrophilic surface results, the monomer–surface parameter, and the anchoring of the microgel to the surfaces (PDF)

## References

[ref1] Gasser U.; Weeks E. R.; Schofield A.; Pusey P.; Weitz D. Real-space imaging of nucleation and growth in colloidal crystallization. Science 2001, 292, 258–262. 10.1126/science.1058457. 11303095

[ref2] Weeks E. R.; Crocker J. C.; Levitt A. C.; Schofield A.; Weitz D. A. Three-dimensional direct imaging of structural relaxation near the colloidal glass transition. Science 2000, 287, 627–631. 10.1126/science.287.5453.627. 10649991

[ref3] Hallett J. E.; Turci F.; Royall C. P. Local structure in deeply supercooled liquids exhibits growing lengthscales and dynamical correlations. Nat. Commun. 2018, 9, 1–10. 10.1038/s41467-018-05371-6. 30115905 PMC6095888

[ref4] Thorneywork A. L.; Abbott J. L.; Aarts D. G.; Dullens R. P. Two-dimensional melting of colloidal hard spheres. Physical review letters 2017, 118, 158001 10.1103/PhysRevLett.118.158001. 28452525

[ref5] Pelton R. Temperature-sensitive aqueous microgels. Advances in colloid and interface science 2000, 85, 1–33. 10.1016/S0001-8686(99)00023-8. 10696447

[ref6] Stieger M.; Richtering W.; Pedersen J. S.; Lindner P. Small-angle neutron scattering study of structural changes in temperature sensitive microgel colloids. J. Chem. Phys. 2004, 120, 6197–6206. 10.1063/1.1665752. 15267506

[ref7] Schimka S.; Lomadze N.; Rabe M.; Kopyshev A.; Lehmann M.; von Klitzing R.; Rumyantsev A. M.; Kramarenko E. Y.; Santer S. Photosensitive microgels containing azobenzene surfactants of different charges. Phys. Chem. Chem. Phys. 2017, 19, 108–117. 10.1039/C6CP04555C. 27722591

[ref8] Wu C.; Wang X. Globule-to-coil transition of a single homopolymer chain in solution. Physical review letters 1998, 80, 4092 10.1103/PhysRevLett.80.4092. 11177949

[ref9] Saunders B. R.; Vincent B. Microgel particles as model colloids: theory, properties and applications. Advances in colloid and interface science 1999, 80, 1–25. 10.1016/S0001-8686(98)00071-2.

[ref10] Senff H.; Richtering W. Temperature sensitive microgel suspensions: Colloidal phase behavior and rheology of soft spheres. J. Chem. Phys. 1999, 111, 1705–1711. 10.1063/1.479430.

[ref11] Karg M.; Hellweg T. New “smart” poly (NIPAM) microgels and nanoparticle microgel hybrids: Properties and advances in characterisation. Curr. Opin. Colloid Interface Sci. 2009, 14, 438–450. 10.1016/j.cocis.2009.08.002.

[ref12] Agrawal G.; Agrawal R. Stimuli-Responsive Microgels and Microgel-Based Systems: Advances in the Exploitation of Microgel Colloidal Properties and Their Interfacial Activity. Polymers 2018, 10, 418 10.3390/polym10040418. 30966453 PMC6415239

[ref13] Rivas-Barbosa R.; Ruiz-Franco J.; Lara-Peña M. A.; Cardellini J.; Licea-Claverie A.; Camerin F.; Zaccarelli E.; Laurati M. Link between Morphology, Structure, and Interactions of Composite Microgels. Macromolecules 2022, 55, 1834–1843. 10.1021/acs.macromol.1c02171. 35283539 PMC8908736

[ref14] Alsayed A. M.; Islam M. F.; Zhang J.; Collings P. J.; Yodh A. G. Premelting at defects within bulk colloidal crystals. Science 2005, 309, 1207–1210. 10.1126/science.1112399. 15994377

[ref15] Zhang Z.; Xu N.; Chen D. T.; Yunker P.; Alsayed A. M.; Aptowicz K. B.; Habdas P.; Liu A. J.; Nagel S. R.; Yodh A. G. Thermal vestige of the zero-temperature jamming transition. Nature 2009, 459, 230–233. 10.1038/nature07998. 19444211

[ref16] Yunker P. J.; Chen K.; Gratale M. D.; Lohr M. A.; Still T.; Yodh A. Physics in ordered and disordered colloidal matter composed of poly (N-isopropylacrylamide) microgel particles. Rep. Prog. Phys. 2014, 77, 056601 10.1088/0034-4885/77/5/056601. 24801604

[ref17] Philippe A.-M.; Truzzolillo D.; Galvan-Myoshi J.; Dieudonné-George P.; Trappe V.; Berthier L.; Cipelletti L. Glass transition of soft colloids. Phys. Rev. E 2018, 97, 040601 10.1103/PhysRevE.97.040601. 29758608

[ref18] Das M.; Zhang H.; Kumacheva E. Microgels: Old materials with new applications. Annu. Rev. Mater. Res. 2006, 36, 117–142. 10.1146/annurev.matsci.36.011205.123513.

[ref19] Fernandez-Nieves A.; Wyss H.; Mattsson J.; Weitz D. A. Microgel suspensions: fundamentals and applications; Wiley: 2011.

[ref20] Zeng Z.; Liang J.; Yu R.; Liu J.; Cao M.; Wang S.; Xia Y. Programmable Color in a Free-Standing Photonic Microgel Film with Ultra-Fast Response. ACS Appl. Mater. Interfaces 2021, 13, 25563–25570. 10.1021/acsami.1c07099. 34013715

[ref21] Heilemann M.; van de Linde S.; Schüttpelz M.; Kasper R.; Seefeldt B.; Mukherjee A.; Tinnefeld P.; Sauer M. Subdiffraction-Resolution Fluorescence Imaging with Conventional Fluorescent Probes. Angew. Chem., Int. Ed. 2008, 47, 6172–6176. 10.1002/anie.200802376. 18646237

[ref22] Huang B.; Bates M.; Zhuang X. Super-resolution fluorescence microscopy. Annual review of biochemistry 2009, 78, 993–1016. 10.1146/annurev.biochem.77.061906.092014. PMC2835776 19489737

[ref23] Betzig E., Hell S. W., Moerner W. E. The nobel prize in chemistry 2014; Nobel Media AB, 2014.

[ref24] Henriques R.; Griffiths C.; Hesper Rego E.; Mhlanga M. M. PALM and STORM: unlocking live-cell super-resolution. Biopolymers 2011, 95, 322–331. 10.1002/bip.21586. 21254001

[ref25] Bückers J.; Wildanger D.; Vicidomini G.; Kastrup L.; Hell S. W. Simultaneous multi-lifetime multi-color STED imaging for colocalization analyses. Opt. Express 2011, 19, 3130–3143. 10.1364/OE.19.003130. 21369135

[ref26] Conley G. M.; Aebischer P.; Nöjd S.; Schurtenberger P.; Scheffold F. Jamming and overpacking fuzzy microgels: Deformation, interpenetration, and compression. Science advances 2017, 3, e1700969 10.1126/sciadv.1700969. 29062888 PMC5650484

[ref27] Busko D.; Baluschev S.; Crespy D.; Turshatov A.; Landfester K. New possibilities for materials science with STED microscopy. Micron 2012, 43, 583–588. 10.1016/j.micron.2011.10.003.

[ref28] Conley G. M.; Nöjd S.; Braibanti M.; Schurtenberger P.; Scheffold F. Superresolution microscopy of the volume phase transition of pNIPAM microgels. Colloids Surf., A 2016, 499, 18–23. 10.1016/j.colsurfa.2016.03.010.

[ref29] Wöll D.; Flors C. Super-resolution Fluorescence Imaging for Materials Science. Small Methods 2017, 1, 1700191 10.1002/smtd.201700191.

[ref30] Gelissen A. P. H.; Oppermann A.; Caumanns T.; Hebbeker P.; Turnhoff S. K.; Tiwari R.; Eisold S.; Simon U.; Lu Y.; Mayer J.; Richtering W.; Walther A.; Wöll D. 3D Structures of Responsive Nanocompartmentalized Microgels. Nano Lett. 2016, 16, 7295 10.1021/acs.nanolett.6b03940. 27701865

[ref31] Pujals S.; Feiner-Gracia N.; Delcanale P.; Voets I.; Albertazzi L. Super-resolution microscopy as a powerful tool to study complex synthetic materials. Nature Reviews Chemistry 2019, 3, 68–84. 10.1038/s41570-018-0070-2.

[ref32] Scheffold F. Pathways and challenges towards a complete characterization of microgels. Nat. Commun. 2020, 11, 1–13. 10.1038/s41467-020-17774-5. 32887886 PMC7473851

[ref33] Bergmann S.; Wrede O.; Huser T.; Hellweg T. Super-resolution optical microscopy resolves network morphology of smart colloidal microgels. Phys. Chem. Chem. Phys. 2018, 20, 5074–5083. 10.1039/C7CP07648G. 29392265

[ref34] Otto P.; Bergmann S.; Sandmeyer A.; Dirksen M.; Wrede O.; Hellweg T.; Huser T. Resolving the internal morphology of core-shell microgels with super-resolution fluorescence microscopy. Nanoscale Advances 2020, 2, 323–331. 10.1039/C9NA00670B. 36134006 PMC9416983

[ref35] Hoppe Alvarez L.; Eisold S.; Gumerov R. A.; Strauch M.; Rudov A. A.; Lenssen P.; Merhof D.; Potemkin I. I.; Simon U.; Wöll D. Deformation of Microgels at Solid-Liquid Interfaces Visualized in Three-Dimension. Nano Lett. 2019, 19, 8862–8867. 10.1021/acs.nanolett.9b03688. 31642321

[ref36] Hoppe Alvarez L.; Rudov A. A.; Gumerov R. A.; Lenssen P.; Simon U.; Potemkin I. I.; Wöll D. Controlling microgel deformation via deposition method and surface functionalization of solid supports. Phys. Chem. Chem. Phys. 2021, 23, 4927–4934. 10.1039/D0CP06355J. 33620358

[ref37] Purohit A.; Centeno S. P.; Wypysek S. K.; Richtering W.; Wöll D. Microgel paint-nanoscopic polarity imaging of adaptive microgels without covalent labelling. Chemical Science 2019, 10, 10336–10342. 10.1039/C9SC03373D. 32110321 PMC6984396

[ref38] Schnitzbauer J.; Strauss M. T.; Schlichthaerle T.; Schueder F.; Jungmann R. Super-resolution microscopy with DNA-PAINT. Nat. Protoc. 2017, 12, 1198–1228. 10.1038/nprot.2017.024. 28518172

[ref39] Conley G. M. Superresolution Microscopy of PNIPAM Microgels; Ph.D. thesis; University of Fribourg: 2017.

[ref40] Gnan N.; Rovigatti L.; Bergman M.; Zaccarelli E. In silico synthesis of microgel particles. Macromolecules 2017, 50, 8777–8786. 10.1021/acs.macromol.7b01600. 29151620 PMC5688413

[ref41] Ninarello A.; Crassous J. J.; Paloli D.; Camerin F.; Gnan N.; Rovigatti L.; Schurtenberger P.; Zaccarelli E. Modeling microgels with a controlled structure across the volume phase transition. Macromolecules 2019, 52, 7584–7592. 10.1021/acs.macromol.9b01122. 31656322 PMC6812067

[ref42] Conley G. M.; Zhang C.; Aebischer P.; Harden J. L.; Scheffold F. Relationship between rheology and structure of interpenetrating, deforming and compressing microgels. Nat. Commun. 2019, 10, 2436 10.1038/s41467-019-10181-5. 31164639 PMC6547648

[ref43] Given that the error bar on the experimental *R*_g_ is of the order of 10 nm, we choose the numerical value within the experimental interval, finding that the best agreement is at its lowest end.

[ref44] Chremos A.; Horkay F.; Douglas J. F. Influence of network defects on the conformational structure of nanogel particles: From “closed compact” to “open fractal” nanogel particles. J. Chem. Phys. 2022, 156, 094903 10.1063/5.0072274. 35259888 PMC8898093

[ref45] Del Monte G.; Truzzolillo D.; Camerin F.; Ninarello A.; Chauveau E.; Tavagnacco L.; Gnan N.; Rovigatti L.; Sennato S.; Zaccarelli E. Two-step deswelling in the Volume Phase Transition of thermoresponsive microgels. Proc. Natl. Acad. Sci. U. S. A. 2021, 118, e2109560118 10.1073/pnas.2109560118. 34508008 PMC8449345

[ref46] Elancheliyan R.; Del Monte G.; Chauveau E.; Sennato S.; Zaccarelli E.; Truzzolillo D. Role of charge content in the two-step deswelling of Poly (N-isopropylacrylamide)-based microgels. Macromolecules 2022, 55, 7526–7539. 10.1021/acs.macromol.2c00995.

[ref47] Hertle Y.; Hellweg T. Thermoresponsive copolymer microgels. J. Mater. Chem. B 2013, 1, 5874–5885. 10.1039/c3tb21143f. 32261054

[ref48] Keerl M.; Pedersen J. S.; Richtering W. Temperature sensitive copolymer microgels with nanophase separated structure. J. Am. Chem. Soc. 2009, 131, 3093–3097. 10.1021/ja807367p. 19206229

[ref49] Geisel K.; Isa L.; Richtering W. Unraveling the 3D localization and deformation of responsive microgels at oil/water interfaces: a step forward in understanding soft emulsion stabilizers. Langmuir 2012, 28, 15770–15776. 10.1021/la302974j. 22891765

[ref50] Camerin F.; Fernandez-Rodriguez M. A.; Rovigatti L.; Antonopoulou M.-N.; Gnan N.; Ninarello A.; Isa L.; Zaccarelli E. Microgels adsorbed at liquid-liquid interfaces: A joint numerical and experimental study. ACS Nano 2019, 13, 4548–4559. 10.1021/acsnano.9b00390. 30865829

[ref51] Harrer J.; Rey M.; Ciarella S.; Löwen H.; Janssen L. M.; Vogel N. Stimuli-responsive behavior of PNiPAm microgels under interfacial confinement. Langmuir 2019, 35, 10512–10521. 10.1021/acs.langmuir.9b01208. 31304759

[ref52] Bochenek S.; Camerin F.; Zaccarelli E.; Maestro A.; Schmidt M. M.; Richtering W.; Scotti A. In-situ study of the impact of temperature and architecture on the interfacial structure of microgels. Nat. Commun. 2022, 13, 1–12. 10.1038/s41467-022-31209-3. 35768399 PMC9243037

[ref53] Destribats M.; Lapeyre V.; Wolfs M.; Sellier E.; Leal-Calderon F.; Ravaine V.; Schmitt V. Soft microgels as Pickering emulsion stabilisers: role of particle deformability. Soft Matter 2011, 7, 7689–7698. 10.1039/c1sm05240c.

[ref54] Scotti A.; Schulte M. F.; Lopez C. G.; Crassous J. J.; Bochenek S.; Richtering W. How Softness Matters in Soft Nanogels and Nanogel Assemblies. Chem. Rev. 2022, 122, 11675–11700. 10.1021/acs.chemrev.2c00035. 35671377

[ref55] Schulte M. F.; Scotti A.; Brugnoni M.; Bochenek S.; Mourran A.; Richtering W. Tuning the Structure and Properties of Ultra-Low Cross-Linked Temperature-Sensitive Microgels at Interfaces via the Adsorption Pathway. Langmuir 2019, 35, 14769–14781. 10.1021/acs.langmuir.9b02478. 31638406

[ref70] Vialetto J.; Ramakrishna S. N.; Isa L. In-situ imaging of the three-dimensional shape of soft responsive particles at fluid interfaces by atomic force microscopy. Sci. Adv. 2022, 8, eabq2019 10.1126/sciadv.abq2019. 36351021 PMC9645722

[ref56] Karg M.; Pastoriza-Santos I.; Pérez-Juste J.; Hellweg T.; Liz-Marzán L. M. Nanorod-coated PNIPAM microgels: Thermoresponsive optical properties. Small 2007, 3, 1222–1229. 10.1002/smll.200700078. 17487899

[ref57] Pergushov D. V.; Sigolaeva L. V.; Balabushevich N. G.; Sharifullin T. Z.; Noyong M.; Richtering W. Loading of doxorubicin into surface-attached stimuli-responsive microgels and its subsequent release under different conditions. Polymer 2021, 213, 123227 10.1016/j.polymer.2020.123227.

[ref58] Chen R.; Shi J.; Liu C.; Li J.; Cao S. In situ self-assembly of gold nanorods with thermal-responsive microgel for multi-synergistic remote drug delivery. Advanced Composites and Hybrid Materials 2022, 5, 2223 10.1007/s42114-021-00306-0.

[ref59] Dave R.; Randhawa G.; Kim D.; Simpson M.; Hoare T. Microgels and Nanogels for the Delivery of Poorly Water-Soluble Drugs. Mol. Pharmaceutics 2022, 19, 1704 10.1021/acs.molpharmaceut.1c00967. 35319212

[ref60] Olivier N.; Keller D.; Gönczy P.; Manley S. Resolution Doubling in 3D-STORM Imaging through Improved Buffers. PLoS One 2013, 8, e69004 10.1371/journal.pone.0069004. 23874848 PMC3714239

[ref61] Kremer K.; Grest G. S. Dynamics of entangled linear polymer melts: A molecular-dynamics simulation. J. Chem. Phys. 1990, 92, 5057–5086. 10.1063/1.458541.

[ref62] Weeks J. D.; Chandler D.; Andersen H. C. Role of Repulsive Forces in Determining the Equilibrium Structure of Simple Liquids. J. Chem. Phys. 1971, 54, 5237–5247. 10.1063/1.1674820.

[ref63] Warner H. R. Kinetic Theory and Rheology of Dilute Suspensions of Finitely Extendible Dumbbells. Industrial & Engineering Chemistry Fundamentals 1972, 11, 379–387. 10.1021/i160043a017.

[ref64] Soddemann T.; Dünweg B.; Kremer K. A generic computer model for amphiphilic systems. Eur. Phys. J. E 2001, 6, 409–419. 10.1007/s10189-001-8054-4.

[ref65] Lo Verso F.; Pomposo J. A.; Colmenero J.; Moreno A. J. Simulation guided design of globular single-chain nanoparticles by tuning the solvent quality. Soft Matter 2015, 11, 1369–1375. 10.1039/C4SM02475C. 25574662

[ref66] Plimpton S. Fast parallel algorithms for short-range molecular dynamics. J. Comput. Phys. 1995, 117, 1–19. 10.1006/jcph.1995.1039.

[ref67] Rovigatti L.; Šulc P.; Reguly I. Z.; Romano F. A comparison between parallelization approaches in molecular dynamics simulations on GPUs. J. Comput. Chem. 2015, 36, 1–8. 10.1002/jcc.23763. 25355527

[ref68] Lopez C. G.; Scotti A.; Brugnoni M.; Richtering W. The Swelling of Poly (Isopropylacrylamide) Near the θ Temperature: A Comparison between Linear and Cross-Linked Chains. Macromol. Chem. Phys. 2018, 220, 1800421 10.1002/macp.201800421.

[ref69] Stukowski A. Visualization and analysis of atomistic simulation data with OVITO–the Open Visualization Tool. Modell. Simul. Mater. Sci. Eng. 2010, 18, 015012 10.1088/0965-0393/18/1/015012.

