# Supplementary material for: Probing Temperature Responsivity of Microgels and Its Interplay with a Solid Surface by Super-Resolution Microscopy and Numerical Simulations
Source: ACS Nano. 2023 Jan 19;17(3):2067–78. doi: 10.1021/acsnano.2c07569 (PMC9933603; doi:10.1021/acsnano.2c07569)
Supplement: Supplementary file 1 — nn2c07569_si_001.pdf [file nn2c07569_si_001.pdf]

Supplementary Information for:
Probing temperature responsivity of microgels and its interplay with a solid surface by super-resolution microscopy and numerical simulations

Xhorxhina Shaulli,[1,*] Rodrigo Rivas-Barbosa,[2,*] Maxime J. Bergman,[1] Chi Zhang,[1] Nicoletta Gnan,[3,2] Frank Scheffold,[1,†] and Emanuela Zaccarelli[3,2,‡]

[1] *Department of Physics, University of Fribourg, Chemin du Musée 3, 1700, Fribourg, Switzerland*
[2] *Department of Physics, Sapienza University of Rome, Piazzale Aldo Moro 2, 00185 Roma, Italy*
[3] *CNR Institute of Complex Systems, Uos Sapienza, Piazzale Aldo Moro 2, 00185, Roma, Italy*

I. SAMPLE PREPARATION FOR DSTORM

To prepare the hydrophilic surface, the coverslip is previously treated with 3 M KOH and sonicated for 10 min followed by exposure for additional 10 min on a UV ozone oven. After treatment the contact angle is measured, as shown in Fig. S1(a). For the hydrophobic surface, the coverslip is first cleaned with 3 M KOH and then exposed overnight to 0.1 mL Hexamethyldisilazane (HMDS). The contact angle is measured after treatment, as reported in Fig. S1(b).

In order to perform a dSTORM experiment on individual microgels, the particles have to be spaced from one another and fixed on the surface. To this aim, we dilute the microgel solution and place around 7 µL between two coverslips in order to create a thin layer and put it to dry shortly at 55 °C. The immobilized particles are then resuspended in a buffer solution containing β-Mercaptoethylamine (Sigma-Aldrich) at concentration 50 mM and pH is adjusted to 8 using 0.1 M HCL. Commonly the buffer solution may require also an oxygen scavenger system, but to keep the system as simple as possible we avoid this and simply make sure to seal the coverslip completely with Picodent glue (Twinsil).

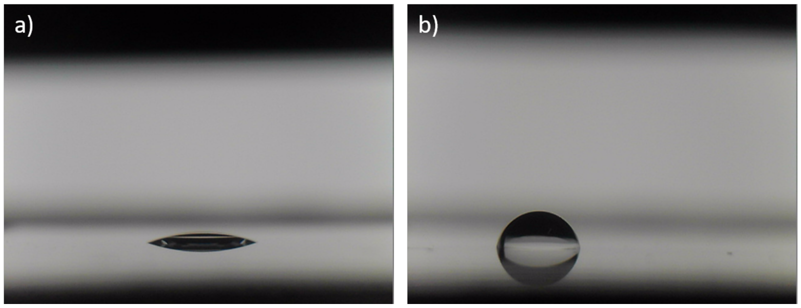

FIG. S1: Images of 4 µL water droplets on the different surfaces for the determination of the contact angle. (a) Contact angle measured on the hydrophilic surface, angle $< 20°$. (b) Contact angle measured on the hydrophobic surface, angle $> 80°$.

II. IMAGE ANALYSIS

The raw data are extracted from the microscope as files with .hdf5 extension, which can be read with several different open source software to reconstruct the super resolved image. In the present case we use Picasso software for processing and post-processing of the data, as it is fast and easy to use.[1] Picasso has several components. The first one we use to reconstruct the image is Localize. The following camera settings are added in the main window: photo-electrons per A/D count = 5 (Sensitivity), base level = 55, EM gain =300, pixel size = 110 nm. In each frame the single molecule spots are identified and fitted using the Maximum

---

* Equal first author
† Equal senior author; frank.scheffold@unifr.ch
‡ Equal senior author; emanuela.zaccarelli@cnr.it

Likelihood Estimation (MLE). The spots are found by considering the net gradient towards the bright center. Then a box of 7 pixels is drawn around it and the exact center is fitted. This is a localisation. For each spot fitted with MLE, the Cramer-Rao lower bound (CRLB) is estimated, the square root of which gives us the localization precision. The second step is image post-processing. Here, we first use the Filter component. We filter the data excluding all the selected spots that have low amount of photon counts or very high photon counts which probably come from more than one fluorophore blinking at the same time, ellipticity higher than 0.2 and localization precision higher than 0.20 pixel in x,y. The last step is rendering. Each localization is shown in Render, with possible smoothing effects. We use individual localization precision in all images reported in the paper, unless otherwise stated. A very important post-processing step is the drift correction. In Render we use the localization-events-based drift correction where images according to their appearance in the movie are split into segments of 1000. Further post-processing tools like Average and Pick are used when stated.

For each experimental data set, only particles with enough localisations are kept.All the picked particles are assigned random colors when reopened in Render. Reconstructed dSTORM images for microgels sitting on hydrophilic and hydrophobic surface are shown in In Fig. S3 and Fig. S4, respectively.

The histograms of the 2D gyration radius R_g are shown in Fig S5 for different experimental data sets.

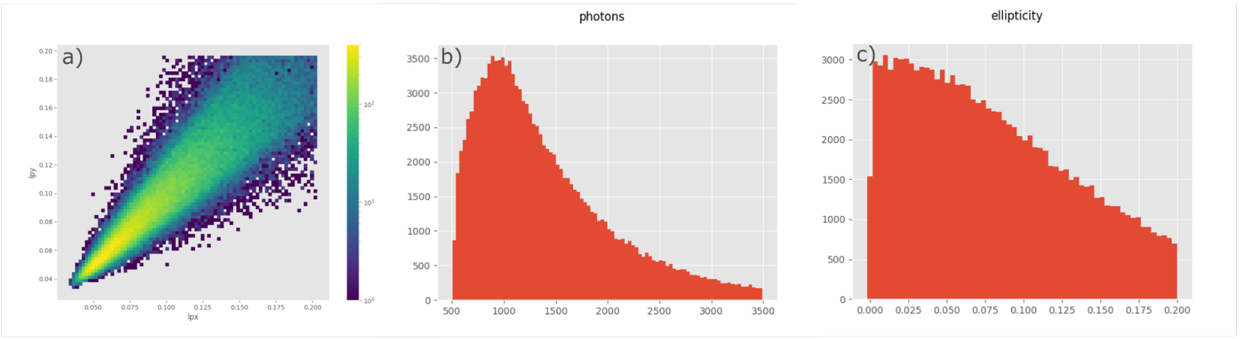

FIG. S2: a) 2D histogram of the localization precision in x and y direction, in camera pixels, as estimated by the Cramer-Rao Lower Bound of the Maximum Likelihood fit. b) Filtered photon count distribution from one experimental data set. c) Filtered ellipticity distribution from one experimental data set.

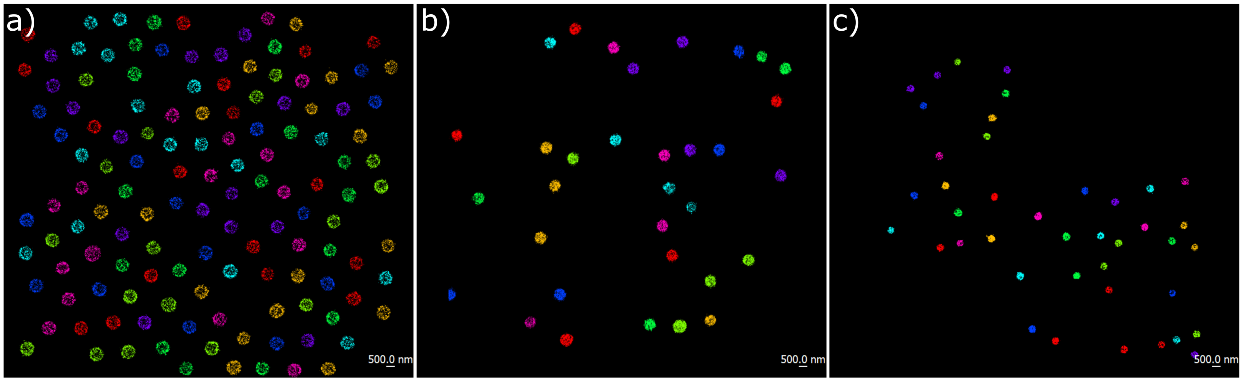

FIG. S3: dSTORM images of microgels on a hydrophilic interface for different temperatures a) 25 °C, b) 30 °C, c) 35 °C.

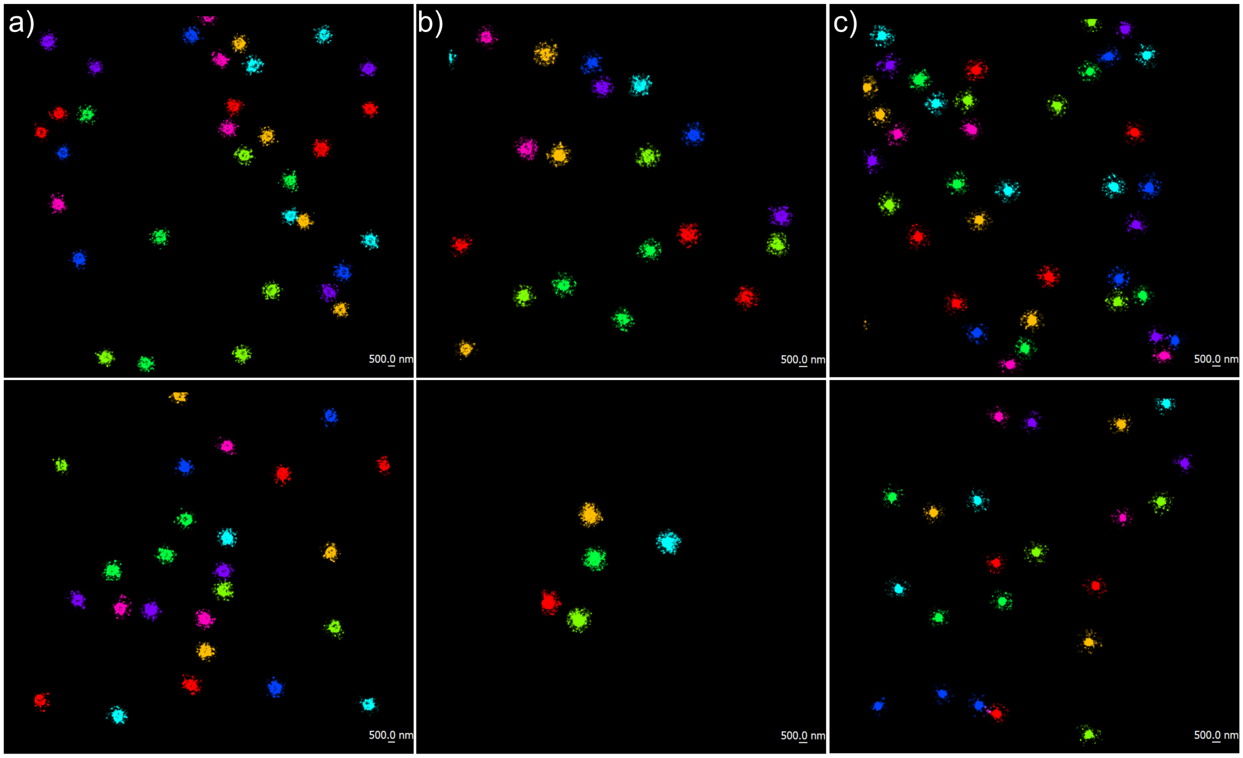

FIG. S4: dSTORM images of microgels on a hydrophobic interface for different temperatures a) 25 °C, b) 30 °C, c) 35 °C.

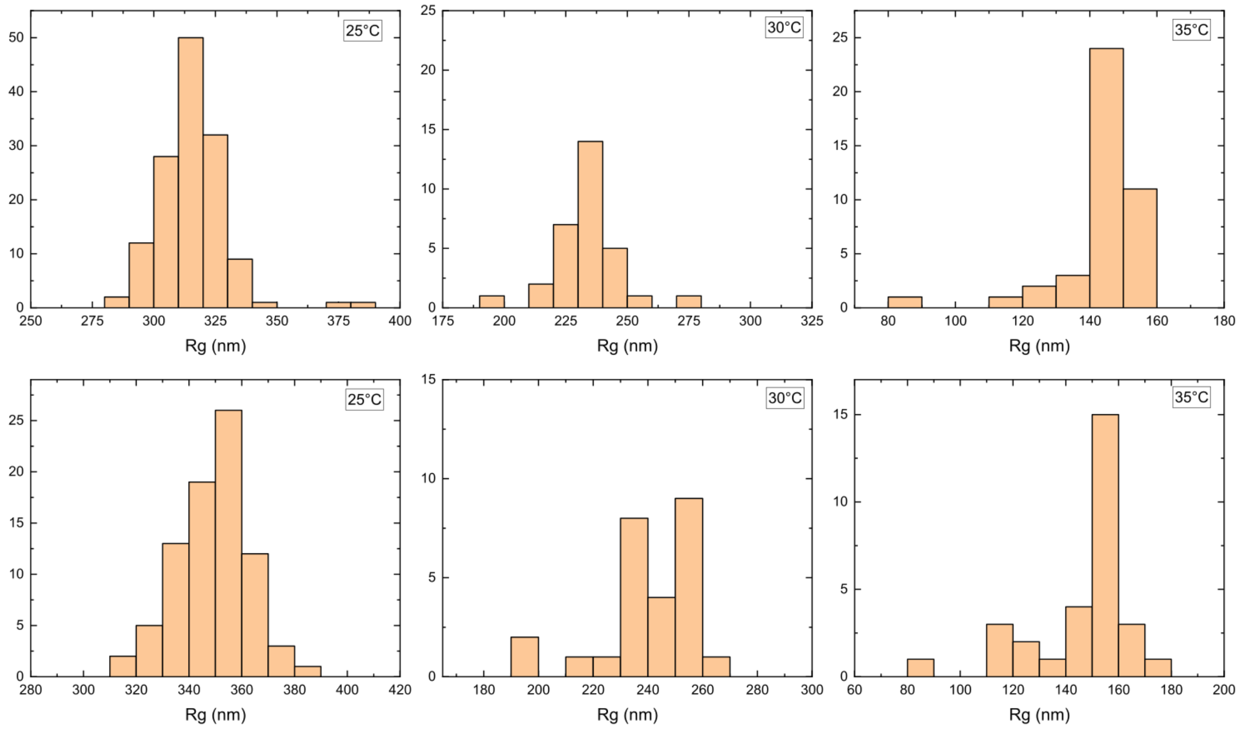

FIG. S5: Experimental 2D R_g Histograms for microgels at 25, 30 and 35 °C. Hydrophilic interface (top panel) and hydrophobic interface (bottom panel)

III. FIT MODEL - HYDROPHILIC INTERFACE

The 2D projected density profiles as obtained from dSTORM experiments are compared to calculated density profiles. The microgels adsorbed to a hydrophilic surface retain their original, spherical swollen shape. In order to emulate the radially decaying density of the microgel under swollen conditions, the classical fuzzy sphere model predicts [2, 3].

$$\rho_{3D}(X,Y,Z) = \rho_{3D}(r) = \text{erfc}\left[\frac{r-R}{\sqrt{2}\sigma_{surf}}\right]/2, \tag{1}$$

where r is the distance from the microgel center of mass, R indicates the core radius and σ_{surf} is the half-width of the fuzzy corona. Because the microgels contain fluorophores predominantly located in their outer shell, their denser core can be considered as 'invisible' in the microscopy videos. We could adapt the fuzzy sphere model to account for the fluorophore profile contained within the microgel using the following expression $2\rho_{3D}(r) = \text{erfc}\left[(r-R)/(\sqrt{2}\sigma)\right]\tanh\left[(r-R_{\text{hole}})/(\sqrt{2}\sigma_{\text{hole}})\right]$ introducing $R_{\text{hole}} < R$ and σ_{hole} as additional fit-parameters. However, we find that a Gaussian ring profile yields a fit of similar good quality

$$\rho_{3D}(r) = \frac{1}{\sigma_{surf}\sqrt{2\pi}}\exp\left[-\left(\frac{r-R}{\sqrt{2}\sigma_{surf}}\right)^2\right]. \tag{2}$$

In this way, the microgel is hollow, but the transition between shell and core is not sharp and the long-distance decay of the density profile is preserved. In order to confidently compare to the experimental profiles, the 3D density profiles are projected in 2D, integrating over Z. Next, the projected 2D profiles are convolved with a Gaussian 2D filter using the experimental resolution in order to include the smearing of the experiment. The theoretical density profiles have been fitted manually to the experimental data. As the hollow core is not the main focus of our study, we concentrate less on fitting the first part of the curve which represents the smooth transition between core and shell, and more on precisely fitting the shell peak and the tail which is sufficient to give us the correct R_{tot} as shown in fig. S6. We finally note that, for 38 °C, the presence of the hollow core is not visible any more and we fit the data using Eq. (1) (with $\sigma_{surf} \sim 0$).

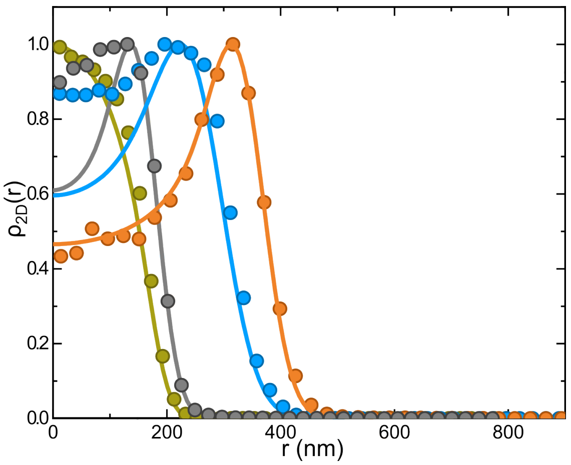

FIG. S6: dSTORM analysis of the microgel radial density profiles illustrating measured 2D profiles (symbols) and corresponding fits with a Gaussian ring or a fuzzy sphere model (lines), as discussed in the text, for microgels at 25 °C (orange), 30 °C (blue), 35 °C (gray) and 38 °C (green). To faciliate comparison among different temperatures, the plots are normalized by setting the peak value to one.

IV. ADDITIONAL DETAILS ON SIMULATIONS

A. Comparison of 2D and 3D density profiles

In the main text we compare experimental and numerical 2D density profiles. For completeness, here we also report the corresponding 3D density profiles, calculated from simulations, and we also differentiate between fully and partially labeled microgels. Fig. S7 shows the (a) 3D and (b) 2D density profiles of fully and partially labeled swollen microgels ($\alpha_{\rm mm} = 0.0$) in the bulk. Unlike the 3D case, the partially labeled 2D profile is non-zero at short distances, due to the projection of fluorophores into the plane. The oscillations that occur at short distances in the 3D full profile are due to the fact that here we are reporting data for one microgel realization. To this aim, it is worth noting that in the manuscript results are always averaged over three independent topologies. However, the noise at small distances even for a single realization is partially removed from the 2D projection, as visible in Fig. S7(b).

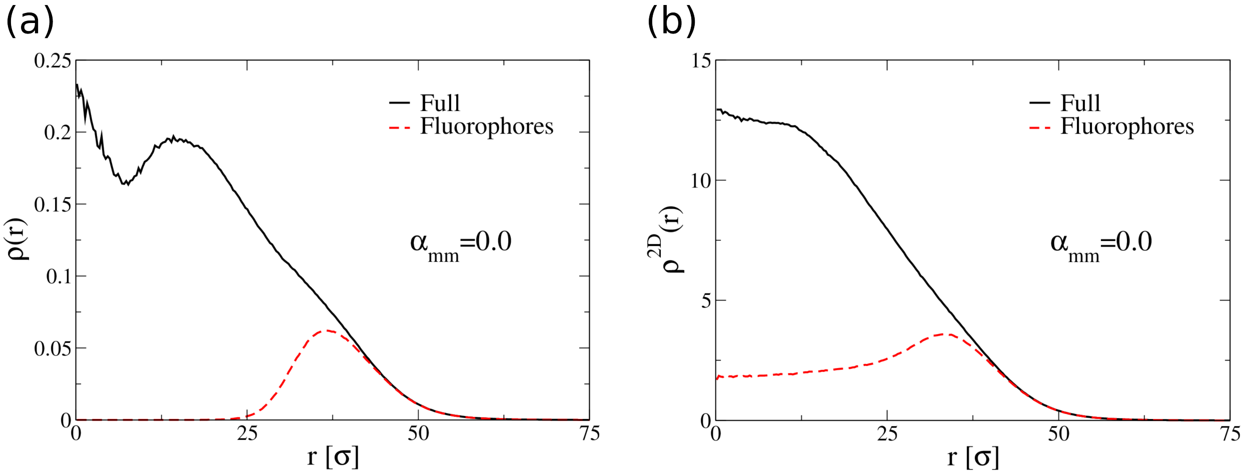

FIG. S7: (a) 3D and (b) 2D density profiles of a fully (solid lines) and partially labeled (dashed lines) swollen microgel in the bulk.

B. Estimating the fluorophore location within the microgels

The 2D experimental and numerical profiles for the hydrophilic/bulk scenario at $T = 25$ °C agree very well without added noise. However, at higher temperature, the same does not hold, as shown in Fig. S8, with deviations increasing with temperature. To this aim, we adopt a Gaussian noise to add to the fluorophore location, as discussed in the main text, with standard deviation $\sigma_{\rm sd}$. The results for different values of $\sigma_{\rm sd}$ are also reported in Fig. S8. Simulation profiles are in best agreement with experimental data close to the hydrophilic surface when the noise parameter is $\sigma_{\rm sd} = 0.0, 0.3, 0.7$ for $T = 25, 30, 35$ °C respectively. Instead, at $T = 38$ °C the experimental data cannot be fitted even with large noise, as shown in Fig. S8(b), but as explained in the main text we find that they can be fitted by considering the full microgel profile (green solid line). This is consistent with the fact that the microgel is fully collapsed at this temperature and the 2D projection 'sees' the microgel as a full, rather than hollow, object.
Taking into account this feature, we analyze again the situation at $T = 35$ °C, for which we find that a large numerical noise ($\sigma_{\rm sd} = 0.7$) is needed to fit the experimental profile. We thus look deeper into each individual experimental profile at this temperature and find that 52% of microgels display a central 2D projected "hole" while the remaining 48% do not. Representative images of these situations are reported in Fig. S9 (b) and (c), respectively. Next, after classifying the microgels in "hole" or "no hole" groups, we fit the averaged experimental profiles of each group separately, as shown in Fig. S9(a). While the "no hole" profile can be fitted with that of a full microgel, the "hole" averaged profile can be described by the fluorophore distribution with added noise $\sigma_{\rm sd} = 0.4$. We then calculate the weigthed average of "hole" and "no hole" profiles (black solid line) with the experimental profile, averaged over all analyzed microgels at this temperature (black circles). This is also compared with the previously found $\sigma_{\rm sd} = 0.7$ fit (dashed gray line) and we can conclude that both descriptions agree quite well with the experimental data. Given that the latter clearly show progressive filling of the hole upon increasing temperature, we thus adopt the description

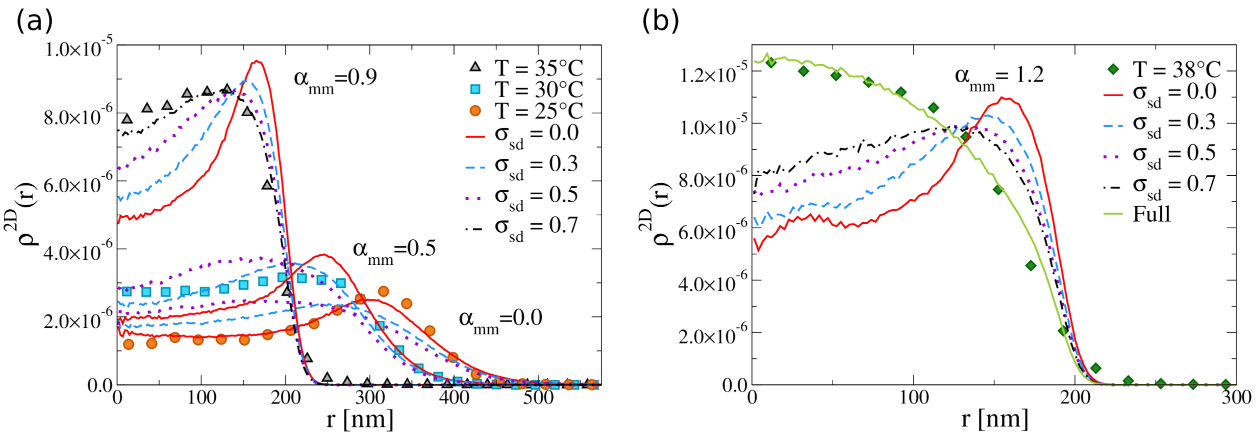

FIG. S8: Experimental 2D density profiles (symbols) at 25, 30, 35 °C compared with numerical data with $\alpha_{mm} = 0.0, 0.5, 0.9$ and varying noise: $\sigma_{\rm sd} = 0.0$ (red solid curves), 0.3 (blue dashed curves), 0.5 (purple dotted curves), and 0.7 (black dash-dotted curves) values. Panel (b) includes the numerical full microgel density profile (solid green) for the 38 °C temperature.

with the two populations of full and hollow microgels in the main text. Hence, the numerical description of the data in Fig. 2 of the main text is the one corresponding to the black solid line in Fig. S9(a). This allows us to have a consistent description of the experiments with increasing temperature and a full microgel profile is then used to fit experiments where no hole is visible, which is the case for $T = 38$ ° C.

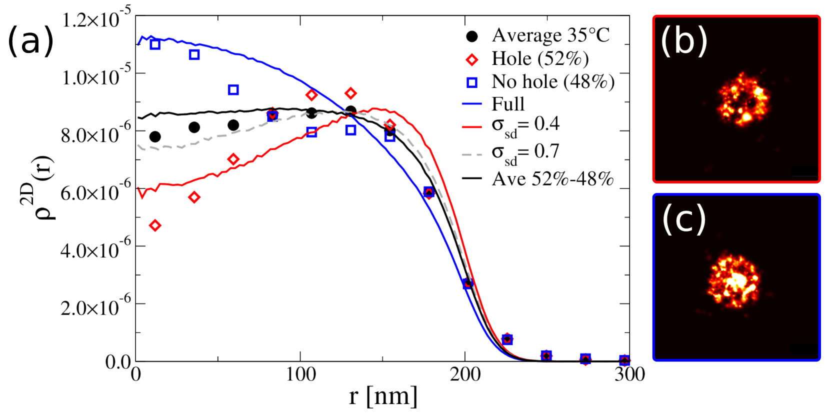

FIG. S9: Experimental 2D density profiles of averaged microgels with projected central hole (red diamonds, 52% of all microgels), no hole (blue squares, 48%), and total average (black filled circles, 100%), and corresponding numerical profiles (lines) with $\alpha_{mm} = 0.9$. The experimental averaged profile can be fitted both with the averaged profile of the hole and no hole fittings (solid black line) and with the noise $\sigma_{\rm sd} = 0.7$ profile (gray dashed line). Side panels: snapshots showing a microgel (b) with and (c) without a hole.

In order to better explain this feature, we report in Fig. S10 the averaged radial distribution of the different fluorophores used to calculate the density profiles when mapped to the swollen state $\alpha_{mm} = 0.0$; in other words, for a microgel at $\alpha_{mm} = 0.0$, we calculated the average radial distribution of the fluorophores used to fit the experimental data at the different temperatures. We also add the whole monomer distribution for comparison. Data are normalized to the same number.
It is clear that, when increasing the noise $\sigma_{\rm sd}$, the distribution broadens and shifts to shorter distances, meaning that when fitting higher temperatures, the fluorophores are seen to be closer the microgels core. However, the addition of the noise is not able to fully reach the limiting full profile case, as shown in Fig. S10, where a small bump in the distribution occurs at small distance, probably due to the presence of the core, which is visible both in the full profile and the one where we mix full and fluorophore profiles (with noise $\sigma_{\rm sd} = 0.4$) as to describe the experimental data at 35 °C. Incidentally, this situation is very similar to the fluorophore profiles with noise $\sigma_{\rm sd} = 0.7$, also fitting such data, with the exception of the previously discussed bump. Finally, it is important to note that, in the case of a hydrophobic surface, all monomers become visible at a lower temperature, namely 35 °C, probably because the full collapse is anticipated by the presence of

the attractive surface, so that also in this case, we fit the experimental data with the full microgel profile and not with the fluorophore list (see also Fig. S15 and related discussion below). These results altogether suggest that, for fully collapsed microgels, the fluorophores appear to be everywhere within the particle, due to the 2D projection of the data. Of course, these considerations about the numerical fitting procedure only apply to the case of partially-labelled microgels and do not affect the main findings of the manuscript, but suggest that for a better and easier comparison to simulations, the dSTORM experiments should be better performed on fully labelled microgels.

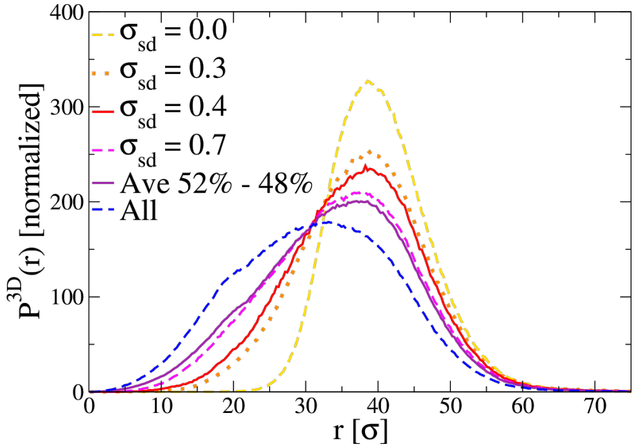

FIG. S10: Averaged radial distributions of the fluorophore lists used to calculate the density profiles. The yellow dashed, orange dotted and pink dashed curves are the distributions calculated from the lists to fit the measurements at 25, 30 and 35 °C respectively, while the blue curve is the distribution of the full microgel, used to fit the 38 °C data. Additionally, the purple solid line is obtained from 52% the red distribution plus 48% the blue one, resulting similar to the pink dashed curve.

C. Bulk vs hydrophilic comparison

We studied the effect of anchoring the microgel to a hydrophilic surface, following the anchoring procedure explained in the Methods. To this aim, we repeat the procedure changing the number of surface-bonded monomers with the values $b = 10, 25, 50, 200, 500, 1000$, in order to explore the influence of surface binding to the density profiles. After anchoring the microgel to the surface and letting the system equilibrate, we perform simulations in hydrophilic conditions, i.e. $\alpha_{\rm ms} = 0.0$, for both swollen ($\alpha_{\rm mm} = 0.0$) and collapsed ($\alpha_{\rm mm} = 0.9$) states. Figs. S11 (a) and (b) show the 2D density profiles at $\alpha_{\rm mm} = 0.0, 0.9$ respectively. While in the swollen state (Fig. S11(a)) the profiles are indistinguishable for all values of b, in the collapsed state (Fig. S11(b)) some differences in the profiles can be seen, although the overall shape is similar for all cases. In particular, we find that the microgel extension is enhanced when increasing b at the cost of a less dense core. This is due to an increasing number of anchored monomers away from the 2D projection of the center of mass: these monomers are restrained from collapsing with the rest of the microgel. Focusing on the short distance profiles in the collapsed state, we find that the optimum value of bonded monomers should be lower than $b < 50$, meaning a number of anchored monomers below 0.1%. Overall, we find that the number of anchored monomers for microgels on hydrophilic surfaces influences the density profiles only at temperatures above the VPT.
Based on the previous results, and to give a complete comparison between the bulk and hydrophilic scenarios, we simulated at all four temperatures (α's) microgels anchored with $b = 25$ bonds. Since we expect no attraction between the microgel and the surface, the interaction between monomers (other than the bonded monomers) and wall particles is exclusively repulsive via the WCA potential. Fig. S12 compares the calculated density profiles of hydrophilic wall-anchored microgels with those simulated in bulk and measured experimentally. The data are in very good agreement at all temperatures.

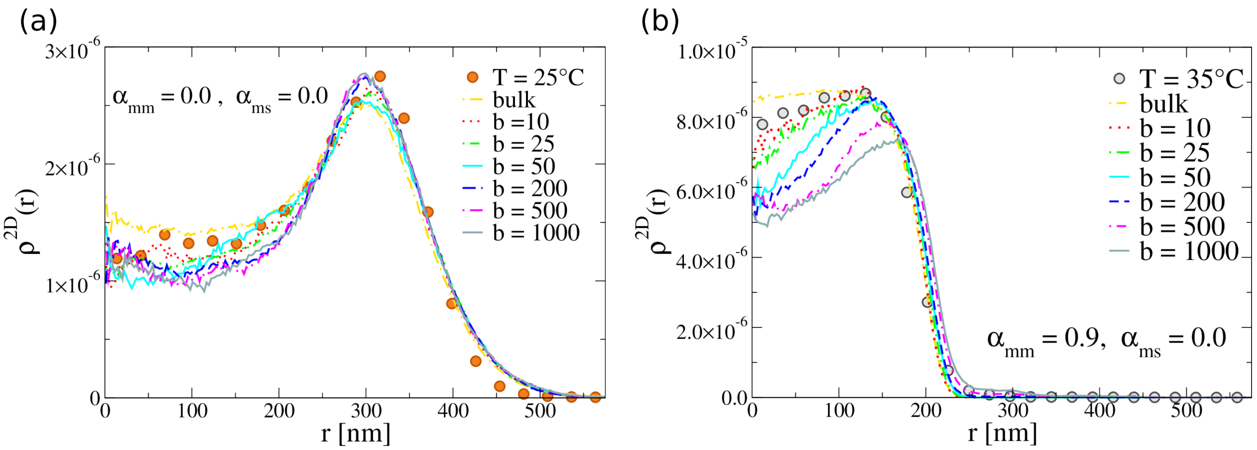

FIG. S11: Experimental density profiles (symbols) and numerical density profiles of a bonded microgels anchored in a swollen state (lines) to a hydrophilic surface using different b number of bonds (a) at 25 °C and (b) 35 °C.

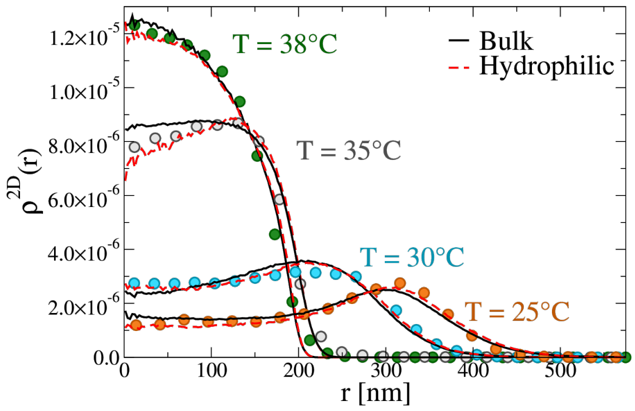

FIG. S12: Experimental 2D density profiles (symbols) compared with numerical profiles of microgels in the bulk (solid black line) and anchored to a hydrophilic surface (red dashed line) with $b = 25$ bonds at 25, 30, 35 and 38 °C.

D. Selection of the monomer-surface attraction parameter

In order to find the optimal value of the monomer-surface attraction parameter $\alpha_{\rm ms}$, which best approximates the hydrophobic HDMS treated surface in experiments, we performed simulations of unbonded microgels in a swollen state ($\alpha_{\rm mm} = 0.0$) near surfaces with different deegres of attraction $\alpha_{\rm ms}$. Figure S13(a) shows the experimental density profiles at 25 °C for the case of the hydrophobic surface compared with the calculated profiles for different $\alpha_{\rm ms}$ values. The simulated density profile with $\alpha_{\rm ms} = 0.9$ is the one which more closely matches the experimental curve: it has a similar height of the peak and tail extension; still, some underestimation at short distances is observed. As discussed in the main text, a better agreement is obtained by adding a fraction of bonded monomers to the surface, to mimic the experimental situation.

Another important feature to take into account is that, with increasing $\alpha_{\rm ms}$, equilibration of the system takes longer and longer. Fig. S13(b) shows again the density profiles but now calculated at short times (from step 5.5×10^6 to 8×10^6) for different degrees of attraction. At this point, we clearly see that the profile with the longest tail does not actually correspond to the most hydrophobic case. This is due to the fact that monomer-surface attached pairs take longer to break, expand and reform, so full equilibration takes a long time.

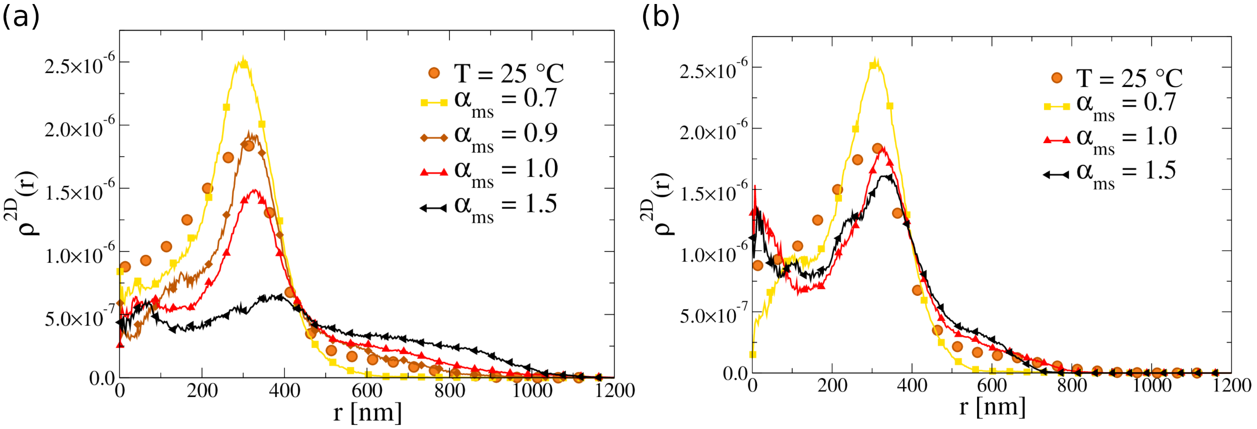

FIG. S13: Experimental 2D density profile at 25 °C (symbols) and numerical 2D density profiles (lines) calculated for an unbonded swollen microgel $\alpha_{\rm mm} = 0.0$ on surfaces at different $\alpha_{\rm ms}$ values at (a) long times and (b) short times (in the window $5.5 \times 10^6 - 8 \times 10^6$ steps). The profile with $\alpha_{\rm ms} = 0.9$ overall gives the best fit of the experimenal data.

E. Effect of the monomer-surface attraction parameter on an anchored microgel

Simulations of an unbonded microgel close to a hydrophobic surface suggested that the monomer-surface parameter should be close to $\alpha_{\rm ms} \sim 0.9$. To verify that this value is correct also for a bonded microgel (with $b = 200$ bonds, as the one used in the main text), we performed additional simulations both for $\alpha_{\rm ms} = 0.9$ and $\alpha_{\rm ms} = 0.8$. Figure S14 shows the 2D density profiles at both values of $\alpha_{\rm ms}$ at $T = 25$ ° C. We find that below the VPT temperature, the data for $\alpha_{\rm ms} = 0.8$ overestimate the peak and underestimate the tail, differently from data for $\alpha_{\rm ms} = 0.9$, confirming that this is the optimal value to best capture the hydrophobicity of the experimental surface, even for anchored microgels.

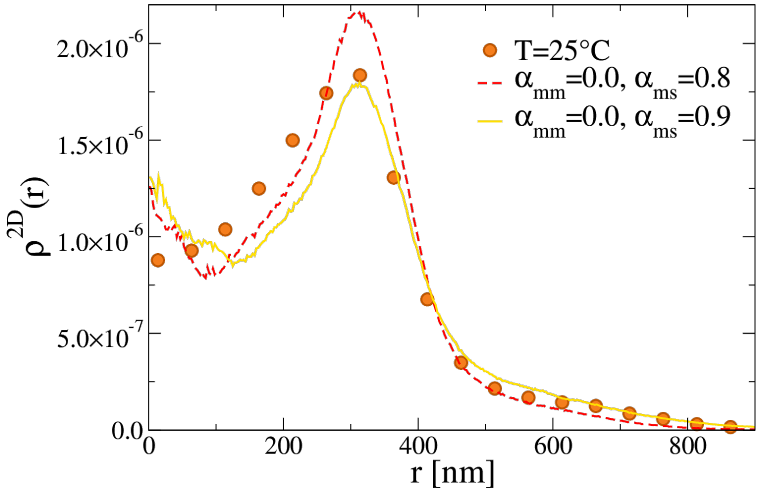

FIG. S14: Experimental density profile T=25 °C (symbols) and numerical density profiles (lines) of the bonded ($b = 200$), swollen microgel ($\alpha_{\rm ms} = 0.0$) close to an attractive surface with $\alpha_{\rm ms} = 0.8$ (dashed red line) and $\alpha_{\rm ms} = 0.9$ (solid yellow line).

F. Effect of the surface selection during the anchoring procedure on microgels above the VPTT for hydrophobic surfaces

We look at the influence of the surface selection during the anchoring procedure on the microgel at high temperatures and close to a hydrophobic surface. The anchoring procedure, explained in the main text, was followed using the main two surfaces $\alpha_{\rm ms} = 0.0, 0.9$; i.e. the microgel was pushed and anchored to

surfaces either completely hydrophilic $\alpha_{\rm ms} = 0.0$ or considerably hydrophobic $\alpha_{\rm ms} = 0.9$; after bonding, the monomer-surface parameter was changed to $\alpha_{\rm ms} = 0.9$ for the first case. During the anchoring on a hydrophobic surface, the microgel expands over it due to the attractive interaction $\alpha_{\rm ms} = 0.9$. This results in an increase of the number of suitable monomers to be anchored whose positions also get further away from the microgels center of mass. After anchoring and simulating above the VPT $\alpha_{\rm mm} = 0.9$, the anchored monomers far away from the microgels center of mass hamper the collapse hence extending the profile. Figure S15 shows the density profiles of the microgel anchored respectively to a hydrophilic and a hydrophobic surface. The profile tail extension is found to be significantly larger when anchoring is done from a hydrophobic surface. The increase of extension is also reflected in a decrease of the profile at shorter distances. Additionally, we show the comparison of the profiles when seen the full microgel or a fraction of its monomers as in the hydrophilic scenario ($\sigma_{\rm sd} = 0.7$). As mentioned in the main text, the peak disappears and the amplitude at short distances increase in the full microgel profiles, resembling closer the experiments. Furthermore, the fitting of the tail extension for the fully seen microgel anchored on a hydrophobic surface captures better the experimental profiles compared the partially labeled one.

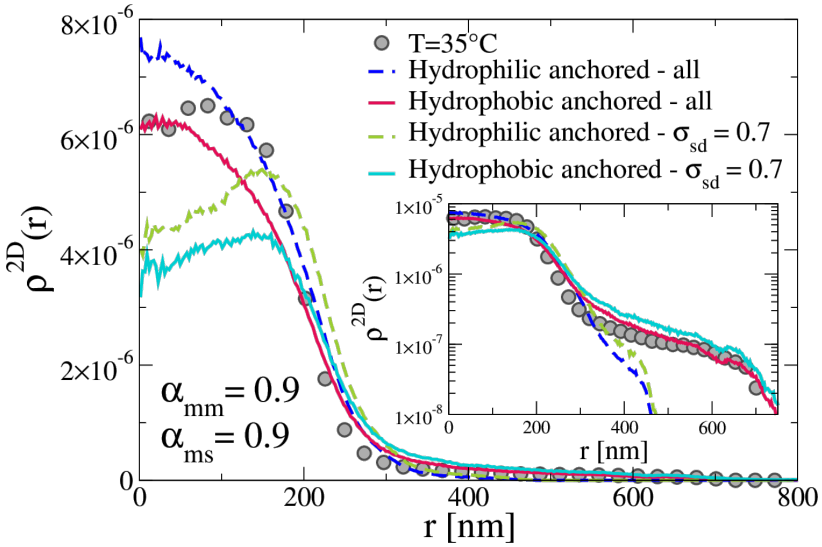

FIG. S15: Experimental density profile (symbols) at 35 °C and numerical density profiles of a bonded microgel anchored in a hydrophilic surface (dashed line) and a hydrophobic surface (solid line) when seeing "all" monomers or a fraction of them following the distributions from Fig. S10. Inset: Logarithmic y-scale to show the density profiles long distance extension.

[1] J. Schnitzbauer, M. T. Strauss, T. Schlichthaerle, F. Schueder, and R. Jungmann, Nature Protocols **12**, 1198 (2017).

[2] M. Stieger, W. Richtering, J. S. Pedersen, and P. Lindner, The Journal of Chemical Physics **120**, 6197 (2004).

[3] G. M. Conley, S. Nöjd, M. Braibanti, P. Schurtenberger, and F. Scheffold, Colloids and Surfaces A: Physicochemical and Engineering Aspects **499**, 18 (2016).
